# Identification of key DNA methylation-driven genes in prostate adenocarcinoma: an integrative analysis of TCGA methylation data

**DOI:** 10.1186/s12967-019-2065-2

**Published:** 2019-09-18

**Authors:** Ning Xu, Yu-Peng Wu, Zhi-Bin Ke, Ying-Chun Liang, Hai Cai, Wen-Ting Su, Xuan Tao, Shao-Hao Chen, Qing-Shui Zheng, Yong Wei, Xue-Yi Xue

**Affiliations:** 1 Department of Urology, The First Affiliated Hospital of Fujian Medical University, 20 Chazhong Road, Fuzhou, 350005 China; 2 Department of Anesthesiology, The First Affiliated Hospital of Fujian Medical University, Fuzhou, 350005 China; 3 Department of Pathology, The First Affiliated Hospital of Fujian Medical University, Fuzhou, 350005 China

**Keywords:** Prostate adenocarcinoma, Methylation, TCGA, GEO, Integrative epigenetic analysis

## Abstract

**Background:**

Prostate cancer (PCa) remains the second leading cause of deaths due to cancer in the United States in men. The aim of this study was to perform an integrative epigenetic analysis of prostate adenocarcinoma to explore the epigenetic abnormalities involved in the development and progression of prostate adenocarcinoma. The key DNA methylation-driven genes were also identified.

**Methods:**

Methylation and RNA-seq data were downloaded for The Cancer Genome Atlas (TCGA). Methylation and gene expression data from TCGA were incorporated and analyzed using MethylMix package. Methylation data from the Gene Expression Omnibus (GEO) were assessed by R package limma to obtain differentially methylated genes. Pathway analysis was performed on genes identified by MethylMix criteria using ConsensusPathDB. Gene Ontology (GO) term enrichment analysis and Kyoto Encyclopedia of Genes and Genomes (KEGG) pathway analysis were also applied for the identification of pathways in which DNA methylation-driven genes significantly enriched. The protein–protein interaction (PPI) network and module analysis in Cytoscape software were used to find the hub genes. Two methylation profile (GSE112047 and GSE76938) datasets were utilized to validate screened hub genes. Immunohistochemistry of these hub genes were evaluated by the Human Protein Atlas.

**Results:**

A total of 553 samples in TCGA database, 32 samples in GSE112047 and 136 samples in GSE76938 were included in this study. There were a total of 266 differentially methylated genes were identified by MethylMix. Plus, a total of 369 differentially methylated genes and 594 differentially methylated genes were identified by the R package limma in GSE112047 and GSE76938, respectively. GO term enrichment analysis suggested that DNA methylation-driven genes significantly enriched in oxidation–reduction process, extracellular exosome, electron carrier activity, response to reactive oxygen species, and aldehyde dehydrogenase [NAD(P)+] activity. KEGG pathway analysis found DNA methylation-driven genes significantly enriched in five pathways including drug metabolism—cytochrome P450, phenylalanine metabolism, histidine metabolism, glutathione metabolism, and tyrosine metabolism. The validated hub genes were MAOB and RTP4.

**Conclusions:**

Methylated hub genes, including MAOB and RTP4, can be regarded as novel biomarkers for accurate PCa diagnosis and treatment. Further studies are needed to draw more attention to the roles of these hub genes in the occurrence and development of PCa.

## Background

Prostate cancer (PCa) remains the second leading cause of deaths due to cancer in the United States in men [1]. Siegel et al. [2] demonstrated that there will have 164,690 newly diagnosed PCa patients and 29,430 deaths in 2018 in the United States. Thus, the diagnosis of PCa in early stage is vitally important [3]. Currently, the serum prostate-specific antigen (PSA) screening remains the primary way for early diagnosis of PCa. However, the sensitivity and specificity of PSA test remains low [4]. Therefore, it is important to find notable biomarkers for the diagnosis of PCa.

Previous studies [5–7] have shown that DNA methylation plays a crucial role in the development and progression of prostate cancer. DNA methylation is treated as a promising investigative tool for the study of progressive prostate cancer because that DNA methylation is a reversible progress [8]. High-throughput screening has been widely used to identify the DNA methylation involved in the initiation and progression of the prostate cancer [9–11]. Epigenetic modifications are crucial for diagnosis and prognosis of prostate cancer and proving additional options for prostate cancer diagnosis and treatment strategies [12, 13].

In this study, prostate cancer associated DNA methylation-driven genes between cancerous and normal samples were identified and GO term enrichment analysis, KEGG pathway analysis, PPI network analysis were also performed respectively.

## Results

### Identification of DNA methylation-driven genes

**Fig. 1:**
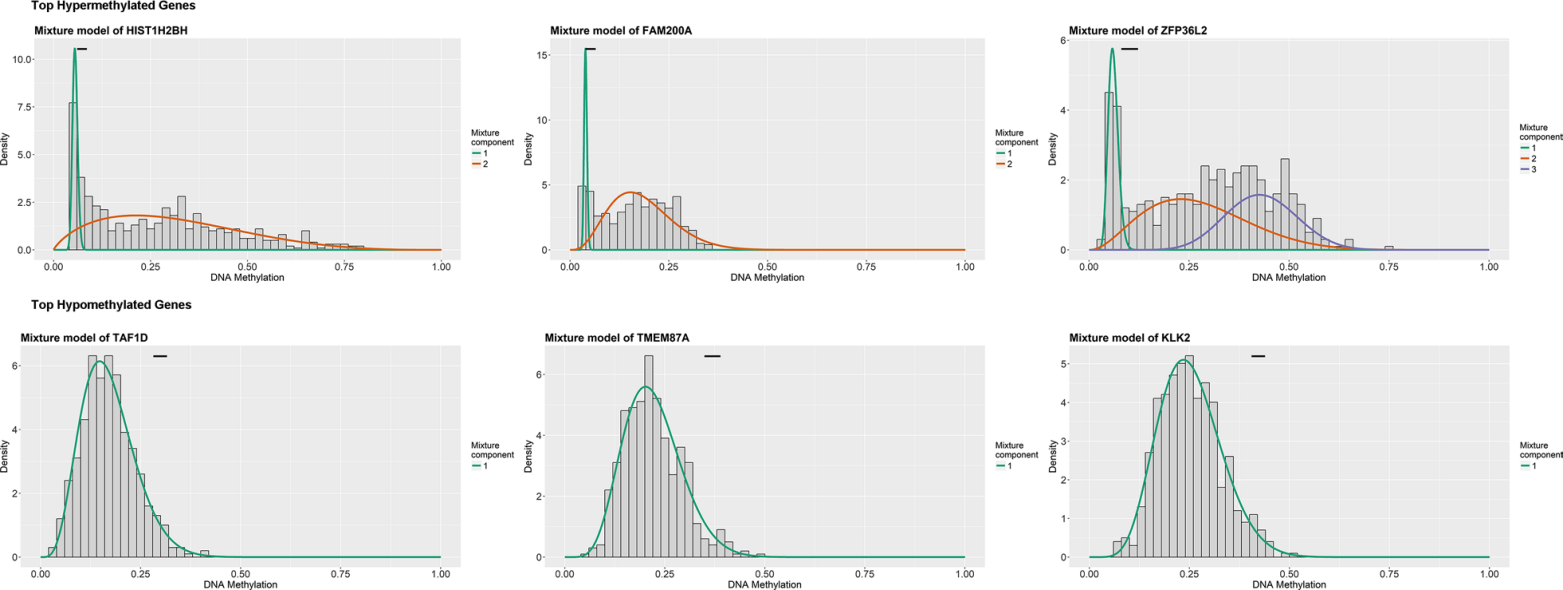
Summary of top hypermethylated and top hypomethylated genes. The horizontal black bar demonstrates the distribution of methylation values in the normal samples (denoted as beta values where higher beta values demonstrate greater methylation). The histogram represents the distribution of methylation in tumor samples

Clinical data of prostate cancer patients extracted from TCGA were demonstrated in Table 1. A total of 266 genes were differentially methylated when comparing tumor to normal by MethylMix criteria for all 553 samples. Representative differential methylation of tumor samples compared with normal samples was demonstrated in Fig. 1 and Table 2. Of these genes, 209 genes (78.57%) were hypermethylated and the remainder of the 57 genes (21.43%) were hypomethylated.

**Table 1:** Clinical dada of prostate cancer patients from TCGA

Characteristic	Total
Cohort size^a^	500
Mean age, years	61.01 ± 6.823
T stage	
pT2a	13 (2.6%)
pT2b	10 (2%)
pT2c	165 (33%)
pT3a	159 (31.8%)
pT3b	136 (27.2%)
pT4	10 (2%)
Unknown	7 (1.4%)
N stage	
N0	348 (69.6%)
N1	79 (15.8%)
Unknown	73 (14.6%)

^a^Three clinical data of prostate cancer patients are not available

**Table 2:** Top 10 hypomethylated genes and hypermethylated genes in patients with prostate cancer

Gene	Normal mean	Tumor mean	Log FC	p value	Adjust p	Cor	Cor p value
Top 10 hypomethylated genes							
TAF1D	0.298851771	0.17046481	− 0.809956148	5.12E−22	1.61E−19	− 0.515133904	3.62E−35
TMEM87A	0.36898334	0.219989007	− 0.746124244	2.40E−22	7.57E−20	− 0.415783216	2.80E−22
KLK2	0.42230197	0.252663444	− 0.741058027	2.34E−25	7.36E−23	− 0.424469057	3.03E−23
MARS	0.661117252	0.407190703	− 0.69920154	2.88E−18	9.08E−16	− 0.402813221	6.87E−21
SLC10A5	0.682378547	0.421046571	-0.696592477	1.19E−16	3.76E−14	− 0.320114016	2.36E−13
KLK3	0.528659515	0.348234132	− 0.602281232	2.38E−22	7.50E−20	− 0.352378214	4.92E−16
MPC2	0.847716806	0.573604393	− 0.563526315	3.24E−22	1.02E−19	− 0.509873152	2.25E−34
ALDH1A3	0.390659331	0.266302408	− 0.552845598	6.96E−20	2.19E−17	− 0.500862763	4.78E−33
CLDN8	0.556942422	0.387056997	− 0.524982157	1.24E−14	3.91E−12	− 0.339347771	6.50E−15
PMEPA1	0.427234317	0.301507194	− 0.50283511	1.93E−21	6.07E−19	− 0.559861766	1.65E−42
Top 10 hypermethylated genes							
HIST1H2BH	0.073047	0.264894	1.858512	1.40E−15	4.41E−13	− 0.37138	9.15E−18
FAM200A	0.052277	0.163374	1.643934	1.78E−18	5.60E−16	− 0.32491	9.86E−14
ZFP36L2	0.101664	0.293078	1.527482	1.59E−13	5.02E−11	− 0.47382	2.72E−29
WFDC2	0.125846	0.347072	1.463575	2.89E−24	9.09E−22	− 0.46874	1.27E−28
SMIM10	0.169555	0.460955	1.44287	1.29E−18	4.06E−16	− 0.52098	4.58E−36
RIPPLY2	0.06161	0.166659	1.435669	4.35E−23	1.37E−20	− 0.33215	2.57E−14
RTP4	0.102519	0.273692	1.416666	4.91E−19	1.55E−16	− 0.51074	1.67E−34
FBXO27	0.048294	0.126663	1.391093	3.13E−12	9.85E−10	− 0.64419	7.67E−60
ZNF492	0.057359	0.147069	1.358401	3.63E−20	1.14E−17	− 0.33472	1.58E−14
HPDL	0.14249	0.356177	1.321732	5.48E−20	1.73E−17	− 0.54417	8.31E−40

**Fig. 2:**
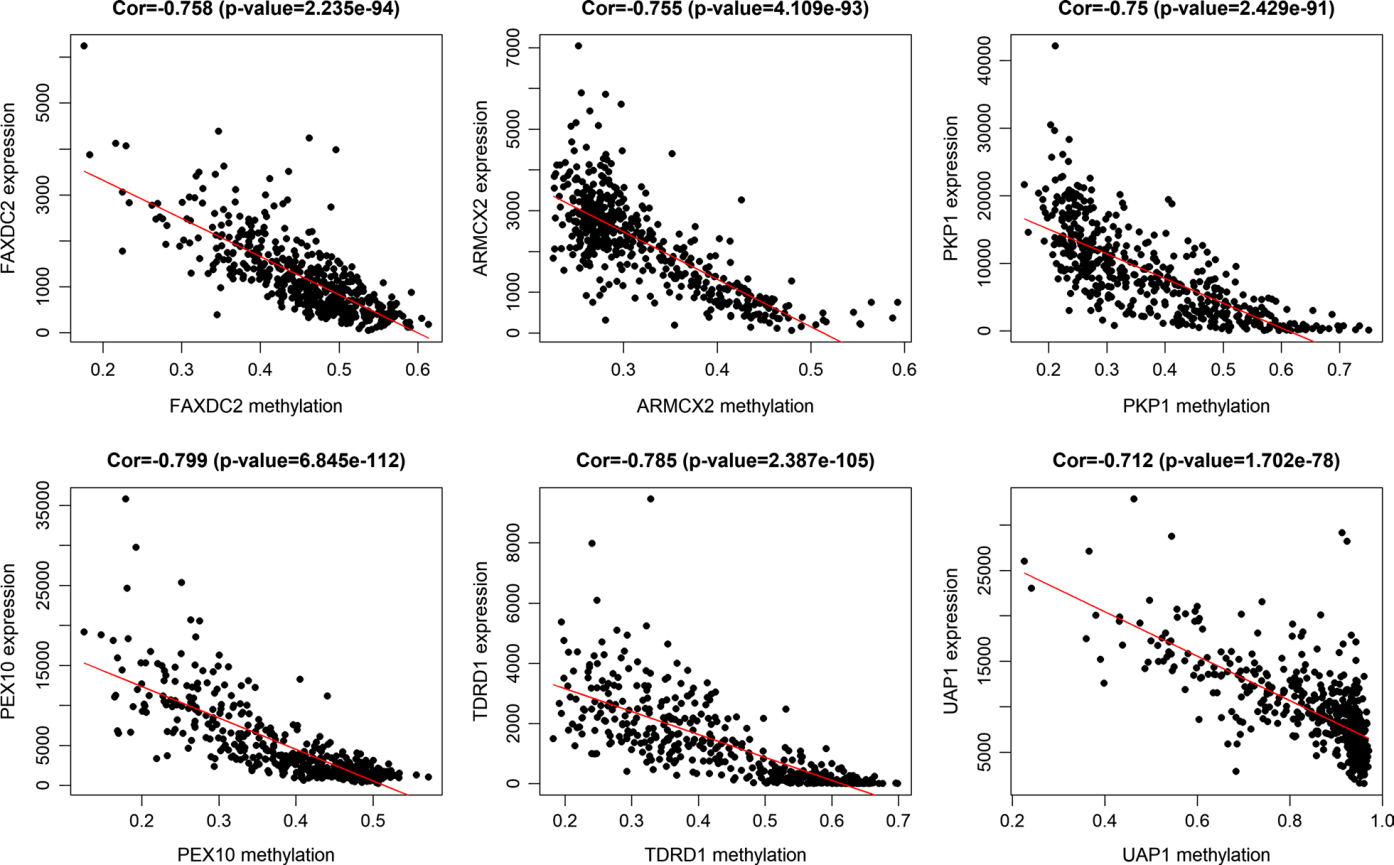
Representative images demonstrated correlation between gene expression and DNA methylation. Gene expression were found to be negatively correlated with DNA methylation. Average β-values are presented on the x-axis, log2 FPKM gene expression values are presented on y-axis

**Fig. 3:**
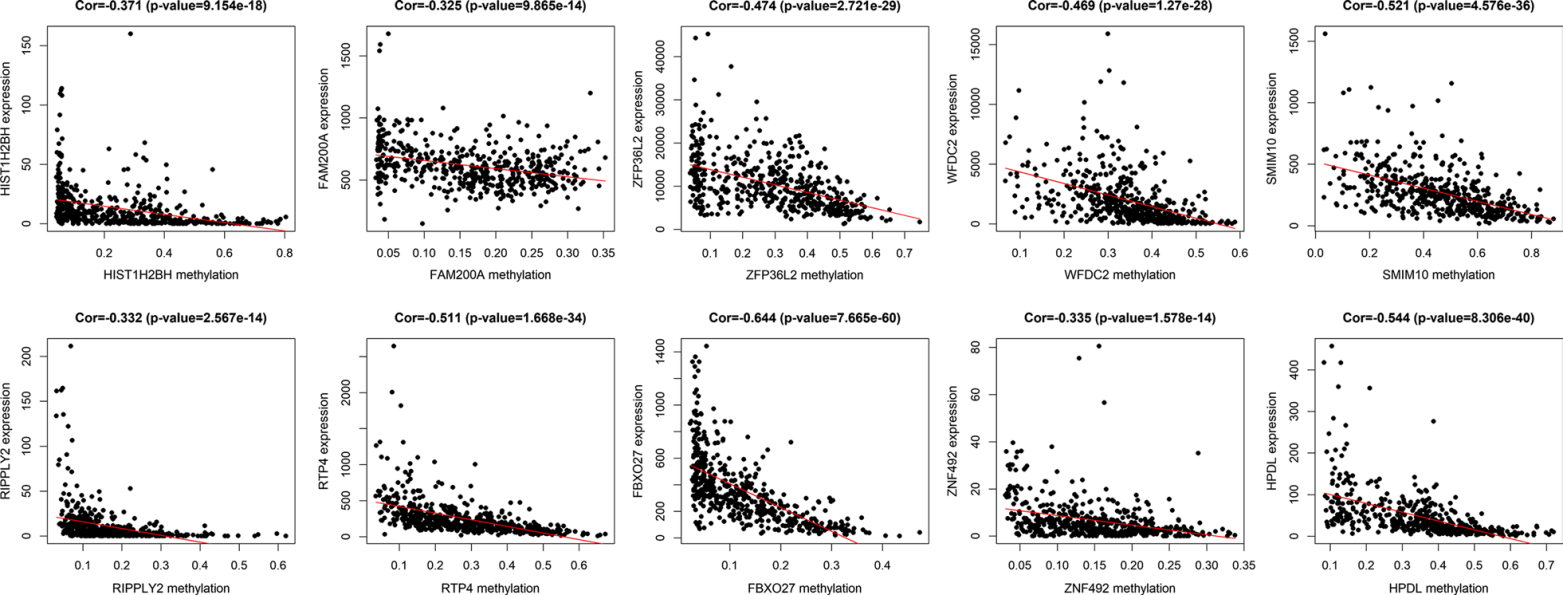
Correlation between genes expression and DNA methylation of top 10 hypermethylated genes. Average β-values are presented on the x-axis, log2 FPKM gene expression values are presented on y-axis

**Fig. 4:**
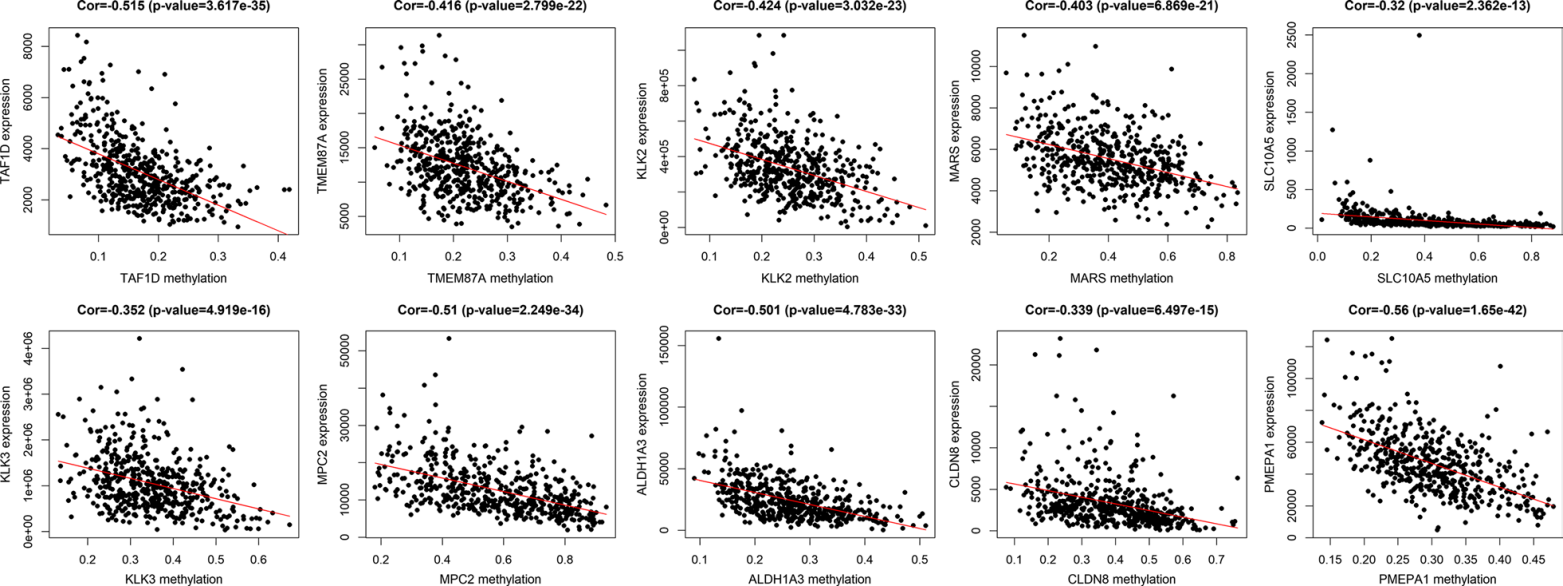
Correlation between genes expression and DNA methylation of top 10 hypomethylated genes. Average β-values are presented on the x-axis, log2 FPKM gene expression values are presented on y-axis

**Fig. 5:**
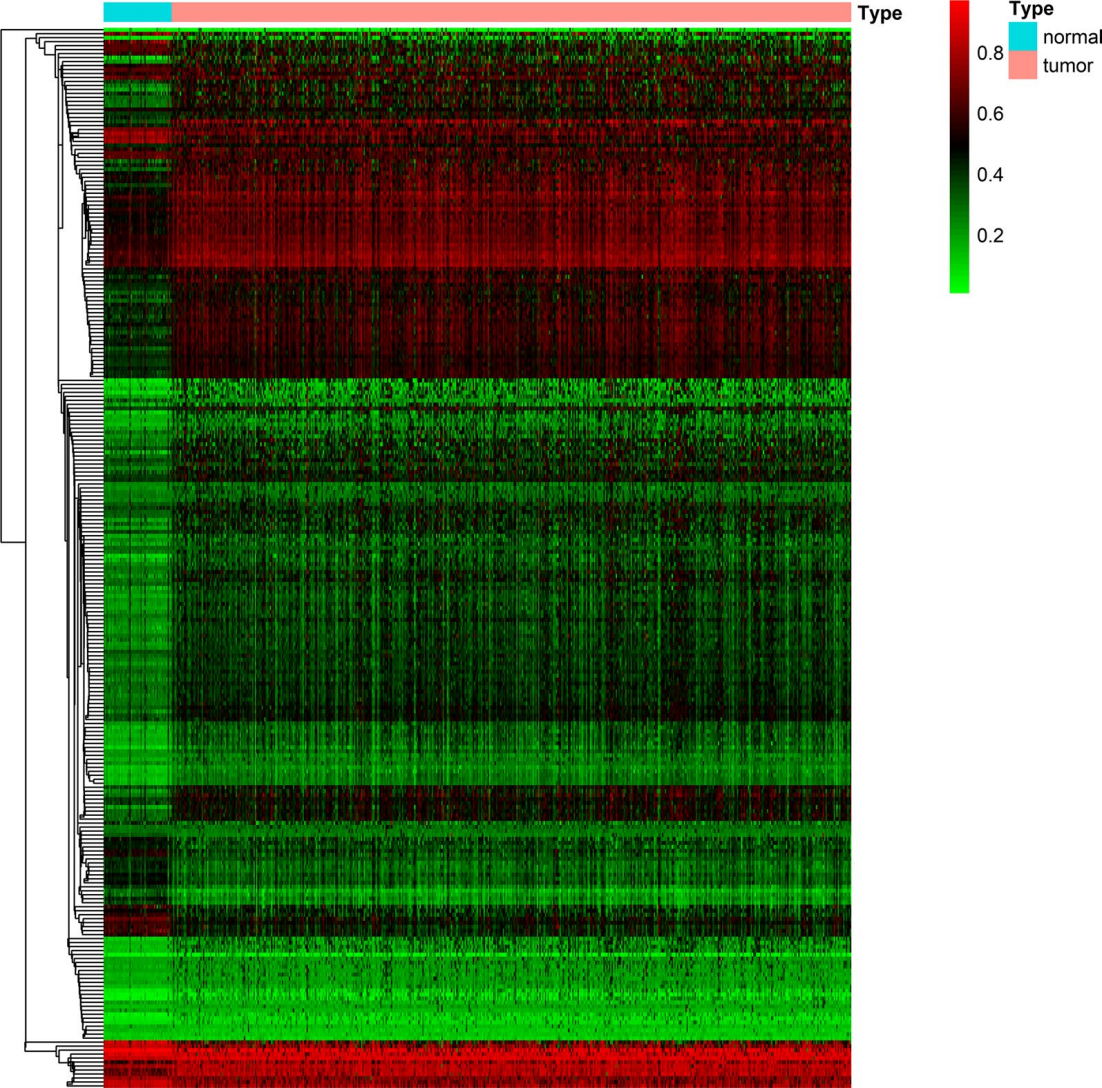
Heatmap of methylation values for 266 genes uniquely methylated in prostate cancer patients

The entire matrix of methylation values was evaluated. In this study, the correlation between gene expression and DNA methylation data was calculated and 266 gene expression were found to be negatively correlated with DNA methylation (Fig. 2). Correlation between genes expression and DNA methylation of top 10 hypermethylated genes (Fig. 3) and top hypomethylated genes (Fig. 4) was also demonstrated. A Heatmap of the methylation values of all patients was demonstrated in Fig. 5.

A total of 369 differentially methylated genes and 594 differentially methylated genes were identified by the R package limma in GSE112047 and GSE76938, respectively.

### Gene ontology terms analysis of DNA methylation-driven genes obtained from TCGA database

**Fig. 6:**
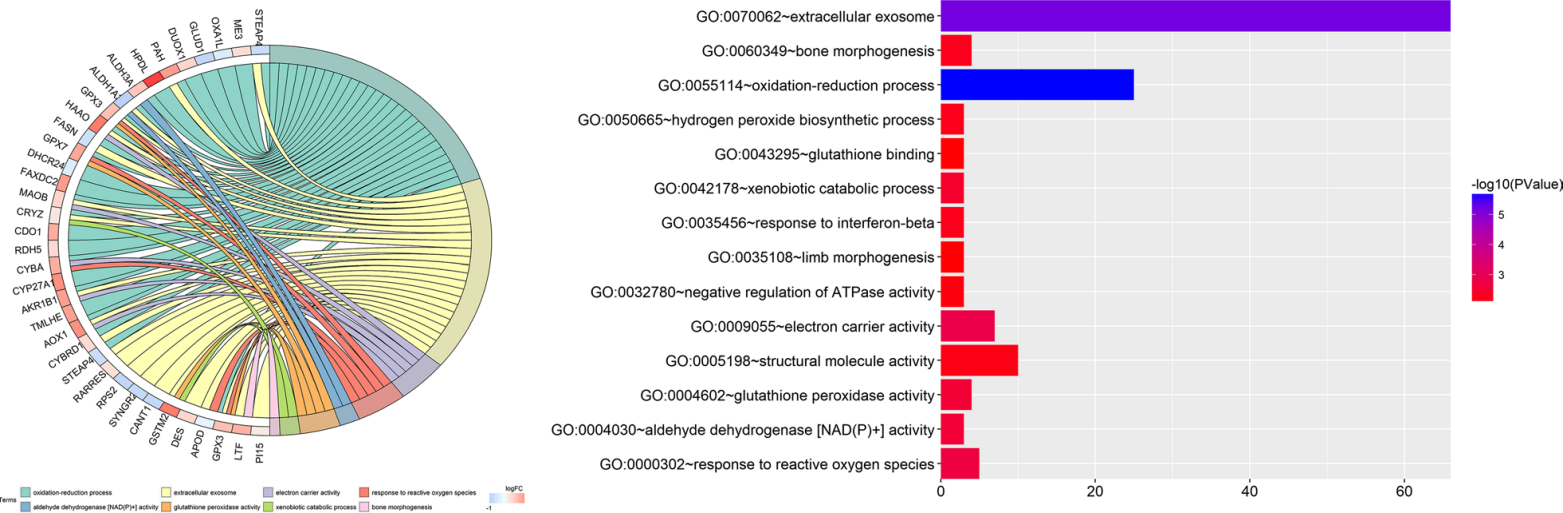
Representative enriched GO terms of 266 DNA methylation driven genes in prostate cancer

GO term enrichment analysis results varied from GO classification. As to biological process, the DNA methylation-driven genes enriched in oxidation–reduction process, response to reactive oxygen species, xenobiotic catabolic process, bone morphogenesis, hydrogen peroxide biosynthetic process, response to interferon-beta, negative regulation of ATPase activity, and limb morphogenesis. For cellular component, the DNA methylation-driven genes enriched in extracellular exosome. For molecular function, the DNA methylation-driven genes enriched in electron carrier activity, aldehyde dehydrogenase [NAD(P)+] activity, glutathione peroxidase activity, structural molecule activity, and glutathione binding (Fig. 6 and Table 3).

**Table 3:** The top 8 GO terms enriched by the 266 DNA methylation driven genes in prostate cancer

Category	Term	Count	p value	Genes
BP	GO:0055114 ~ oxidation–reduction process	25	2.13E−06	STEAP4, ME3, OXA1L, GLUD1, DUOX1, PAH, HPDL, ALDH3A1, ALDH1A3, GPX3, HAAO, FASN, GPX7, DHCR24, FAXDC2, MAOB, CRYZ, CDO1, RDH5, CYBA, CYP27A1, AKR1B1, TMLHE, AOX1, CYBRD1
CC	GO:0070062 ~ extracellular exosome	66	6.38E−06	STEAP4, RARRES2, RPS2, SYNGR2, CANT1, GSTM2, DES, APOD, GPX3, LTF, PI15, ZDHHC1, BST2, CFTR, EEF2, CD40, CD38, KRT17, KRT15, TMEM106A, CYBRD1, HSPB1, SERPINB1, NEU1, CSTA, MFAP4, WFDC2, GSTP1, ACP5, PAH, CD74, B3GNT8, KRT5, ITGB8, KRT7, ALDH1A3, ENTPD5, FASN, RPL3, HAAO, ANGPTL1, RPL7A, MARS, BHMT2, S100A16, LGALS3, KLK2, KLK3, HIST1H2BH, MAOB, KLK1, CRYZ, ANXA2, PROM1, ORM1, ACSM1, C1ORF116, PKP1, PHB2, RAB34, AKR1B1, CAPG, AOX1, ALDH2, SLC46A3, PON3
MF	GO:0009055 ~ electron carrier activity	7	0.001504105	ACOX2, CYBA, AOX1, AKR1B1, MAOB, ALDH2, HAAO
BP	GO:0000302 ~ response to reactive oxygen species	5	0.0019364	CYBA, APOD, GPX3, GPX7, GSTP1
MF	GO:0004030 ~ aldehyde dehydrogenase [NAD(P)+] activity	3	0.002720156	ALDH1A3, ALDH2, ALDH3A1
MF	GO:0004602 ~ glutathione peroxidase activity	4	0.002838234	GSTM2, GPX3, GPX7, GSTP1
BP	GO:0042178 ~ xenobiotic catabolic process	3	0.003748861	GSTM1, GSTM2, CRYZ
BP	GO:0060349 ~ bone morphogenesis	4	0.005820424	SP5, LTF, ACP5, RIPPLY2

### KEGG pathway analysis of DNA methylation-driven genes obtained from TCGA

**Fig. 7:**
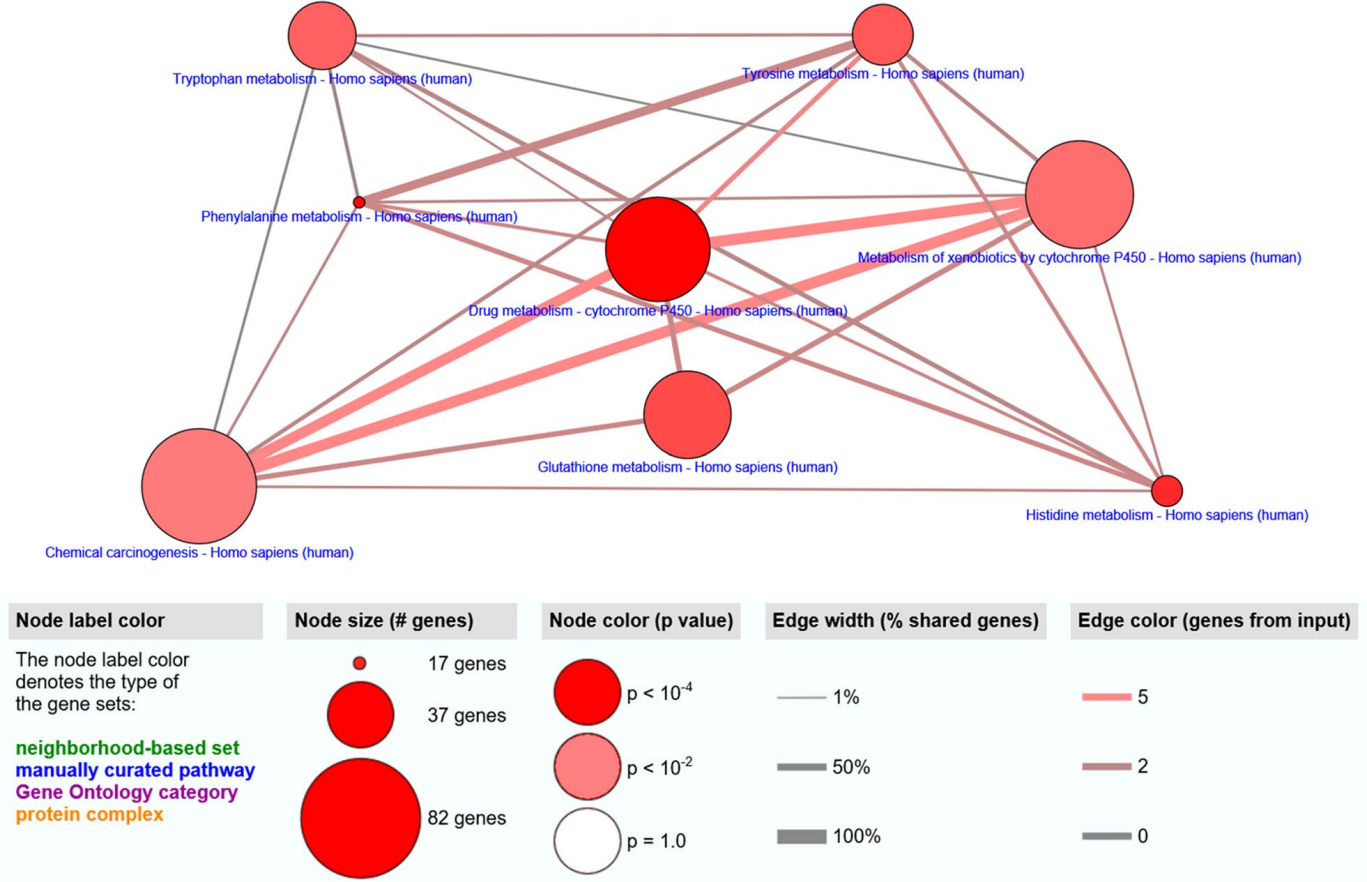
Pathways enriched of 266 DNA methylation driven genes in prostate cancer

KEGG pathway analysis found five significantly enriched pathways. Seven DNA methylation-driven genes enriched in drug metabolism—cytochrome P450. Four DNA methylation-driven genes enriched in Phenylalanine metabolism. Four DNA methylation-driven genes enriched in Histidine metabolism. Five DNA methylation-driven genes enriched in Glutathione metabolism. Four DNA methylation-driven genes enriched in Tyrosine metabolism (Fig. 7 and Table 4).

**Table 4:** Pathway enriched by the 266 DNA methylation driven genes

Pathway	External_id	Members_input_overlap	p-value	q-value	Size
Drug metabolism—cytochrome P450	hsa00982	AOX1; GSTM1; GSTM2; ALDH3A1; MAOB; GSTP1; ALDH1A3	6.59E−05	0.003828899	70
Phenylalanine metabolism	hsa00360	PAH; MAOB; ALDH3A1; ALDH1A3	9.23E−05	0.003828899	17
Histidine metabolism	hsa00340	MAOB; ALDH3A1; ALDH2; ALDH1A3	0.000320403	0.008864472	23
Glutathione metabolism	hsa00480	GSTM1; GSTM2; GPX3; GSTP1; GPX7	0.00114281	0.023713299	54
Tyrosine metabolism	hsa00350	AOX1; ALDH3A1; ALDH1A3; MAOB	0.001836818	0.030491186	36
Tryptophan metabolism	hsa00380	MAOB; HAAO; ALDH2; AOX1	0.002722806	0.037665486	40
Metabolism of xenobiotics by cytochrome P450	hsa00980	GSTM1; GSTM2; ALDH3A1; GSTP1; ALDH1A3	0.00433977	0.051457275	74
Chemical carcinogenesis	hsa05204	GSTM1; GSTM2; ALDH3A1; GSTP1; ALDH1A3	0.007094498	0.073605418	82

### PPI network analysis of DNA methylation-driven genes

**Fig. 8:**
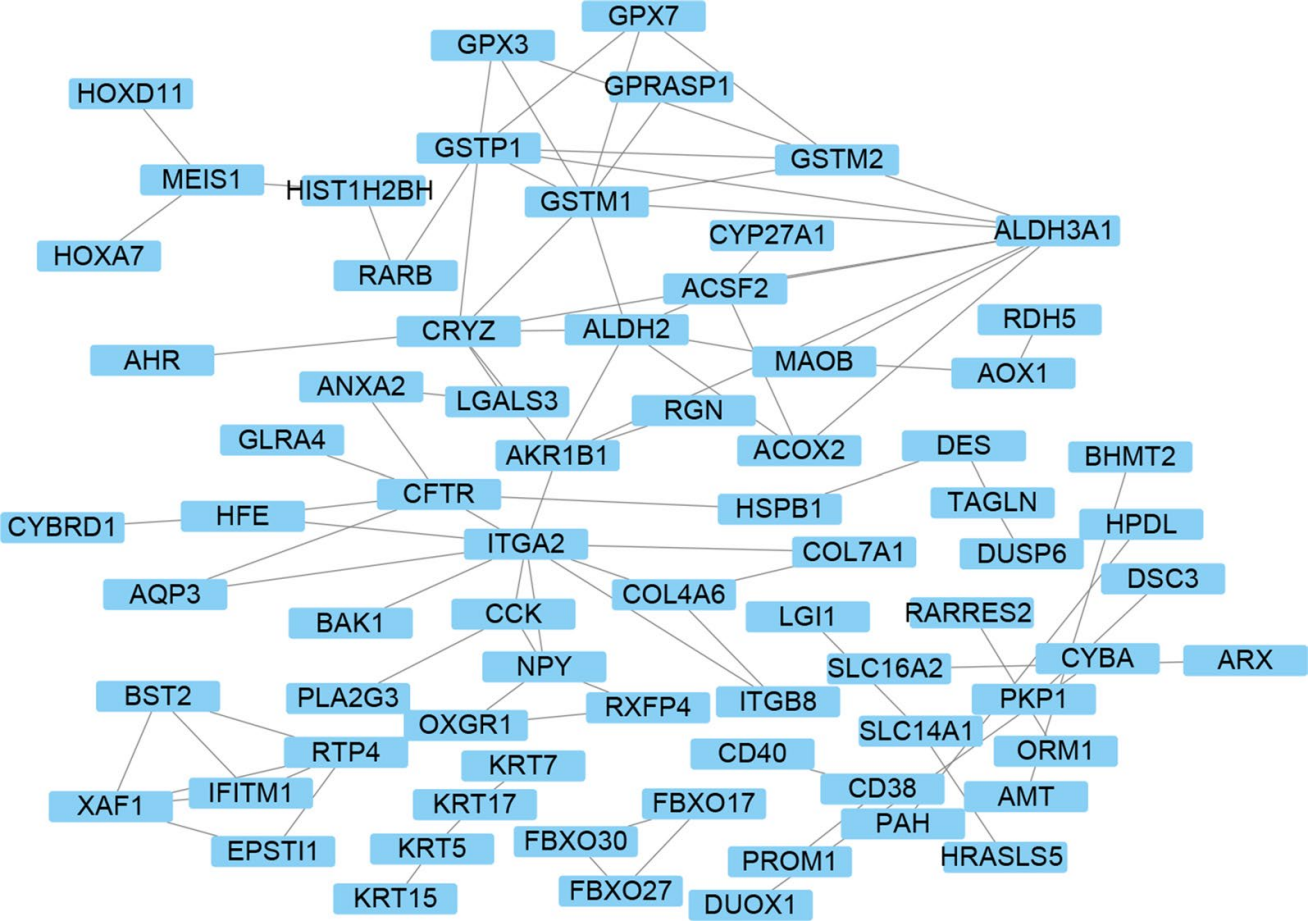
PPI network conducted by 266 DNA methylation driven genes

**Fig. 9:**
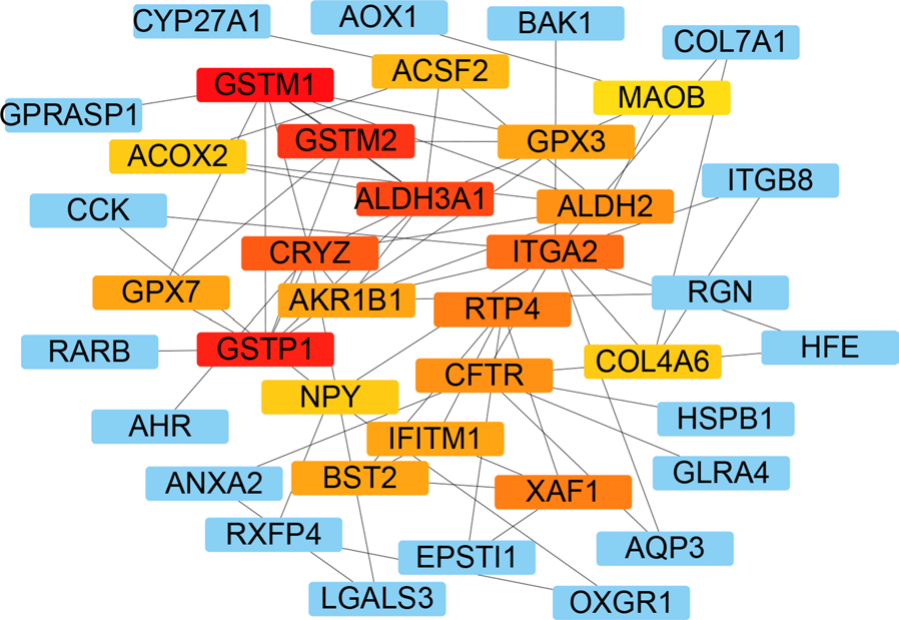
A total of 20 hub genes were found by using the PPI network analysis in Cytoscape software

**Fig. 10:**
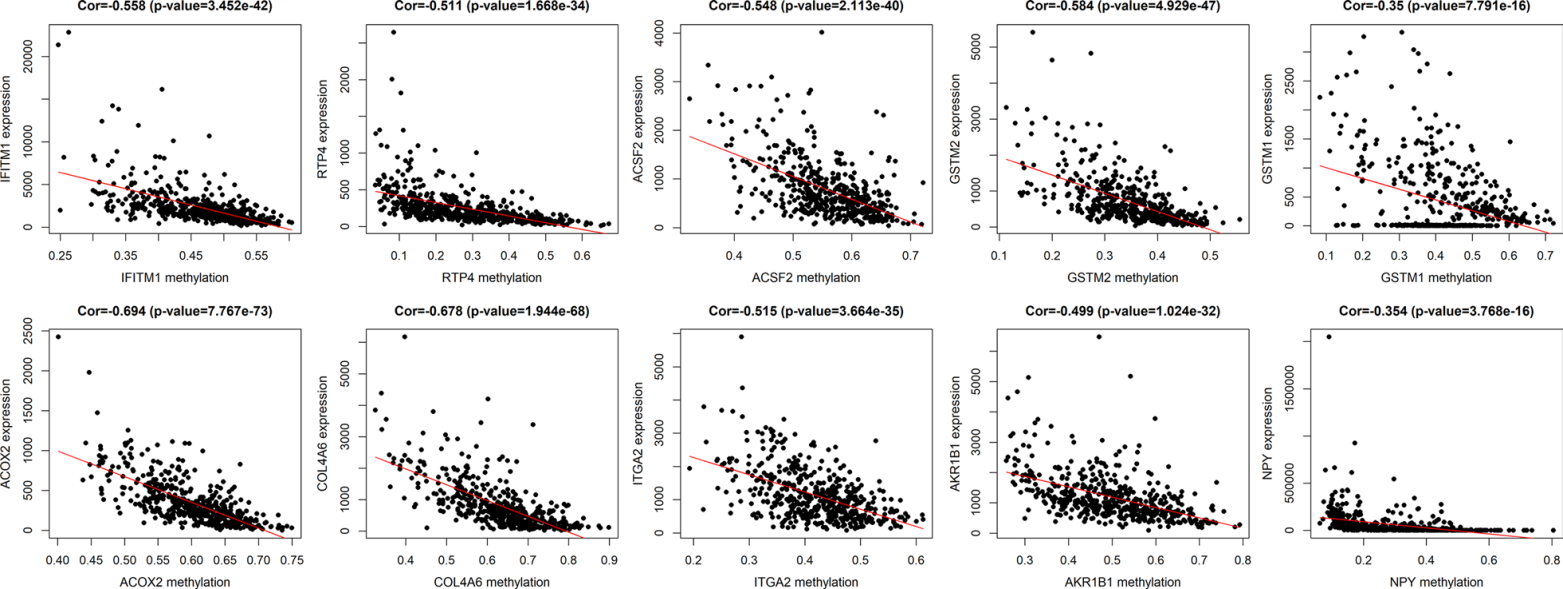
Correlation between genes expression and DNA methylation of top 10 hub genes. Average β-values are presented on the x-axis, log2 FPKM gene expression values are presented on y-axis

PPI network of DNA methylation-driven genes, consisting of 75 nodes and 90 edges, was constructed by Cytoscape software, based on STRING database. The top 20 differentially expressed DNA methylation-driven genes with high degree of connectivity were selected as the hub DNA methylation-driven genes. There hub genes were IFITM1, RTP4, ACSF2, GSTM2, GSTM1, ACOX2, COL4A6, ITGA2, AKR1B1, NPY, CFTR, GPX7, ALDH3A1, CRYZ, ALDH2, MAOB, GSTP1, GPX3, XAF1, and BST2 (Fig. 8), which might play important roles in DNA methylation in prostate cancer patients. CytoHubba was used to carry out the top 20 hub DNA methylation-driven genes in Cytoscape software (Fig. 9). Correlation between genes expression and DNA methylation of top 10 hub genes was also demonstrated in Fig. 10.

### Validation of candidate hub DNA methylation-driven genes by the Gene Expression Omnibus (GEO) database

**Fig. 11:**
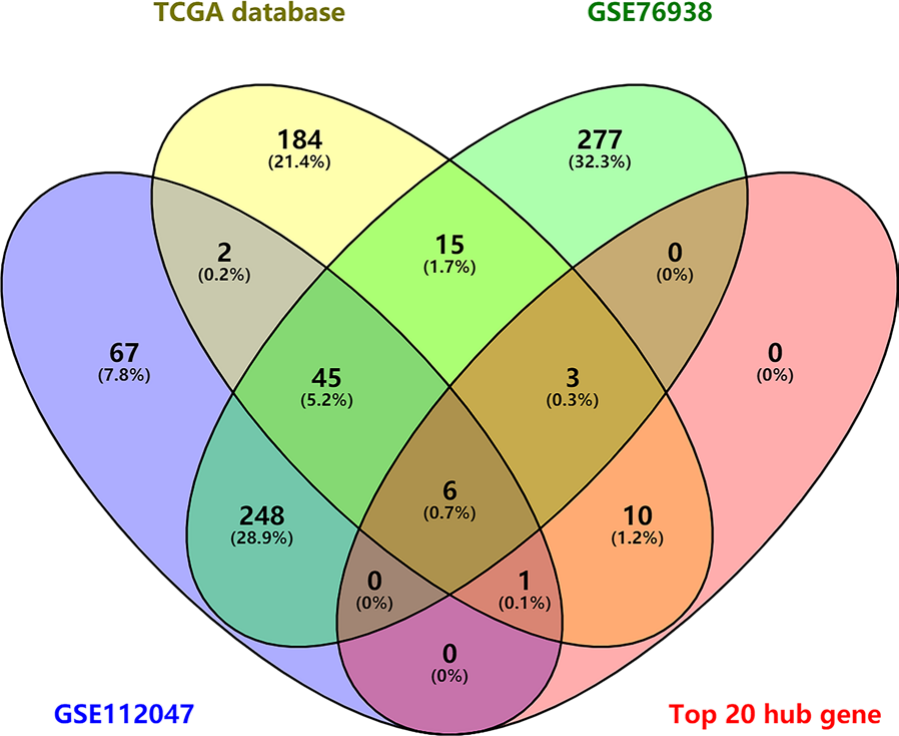
A Venn diagram was used to demonstrate a common list of 6 methylated genes including AKR1B1, RTP4, MAOB, GSTM2, GPX3, and COL4A6

To further investigate the candidate hub DNA methylation-driven genes, GEO database was used to validate these selected genes. Two methylation profile datasets (GSE76938 and GSE112047) were extracted from the GEO for the validation. We then overlapped the differentially methylated genes among 266 genes obtained from TCGA database (Additional file 1: Table S1), 369 genes obtained from GSE112047 (Additional file 2: Table S2), 594 genes obtained from GSE 76938 (Additional file 2: Table S2), and top 20 hub genes obtained from PPI network analysis and identified a common list of 6 methylated genes including AKR1B1, RTP4, MAOB, GSTM2, GPX3, and COL4A6. The outcome was demonstrated by a Venn diagram (Fig. 11).

### Gene ontology terms analysis of DNA methylation-driven genes obtained from GEO database

**Fig. 12:**
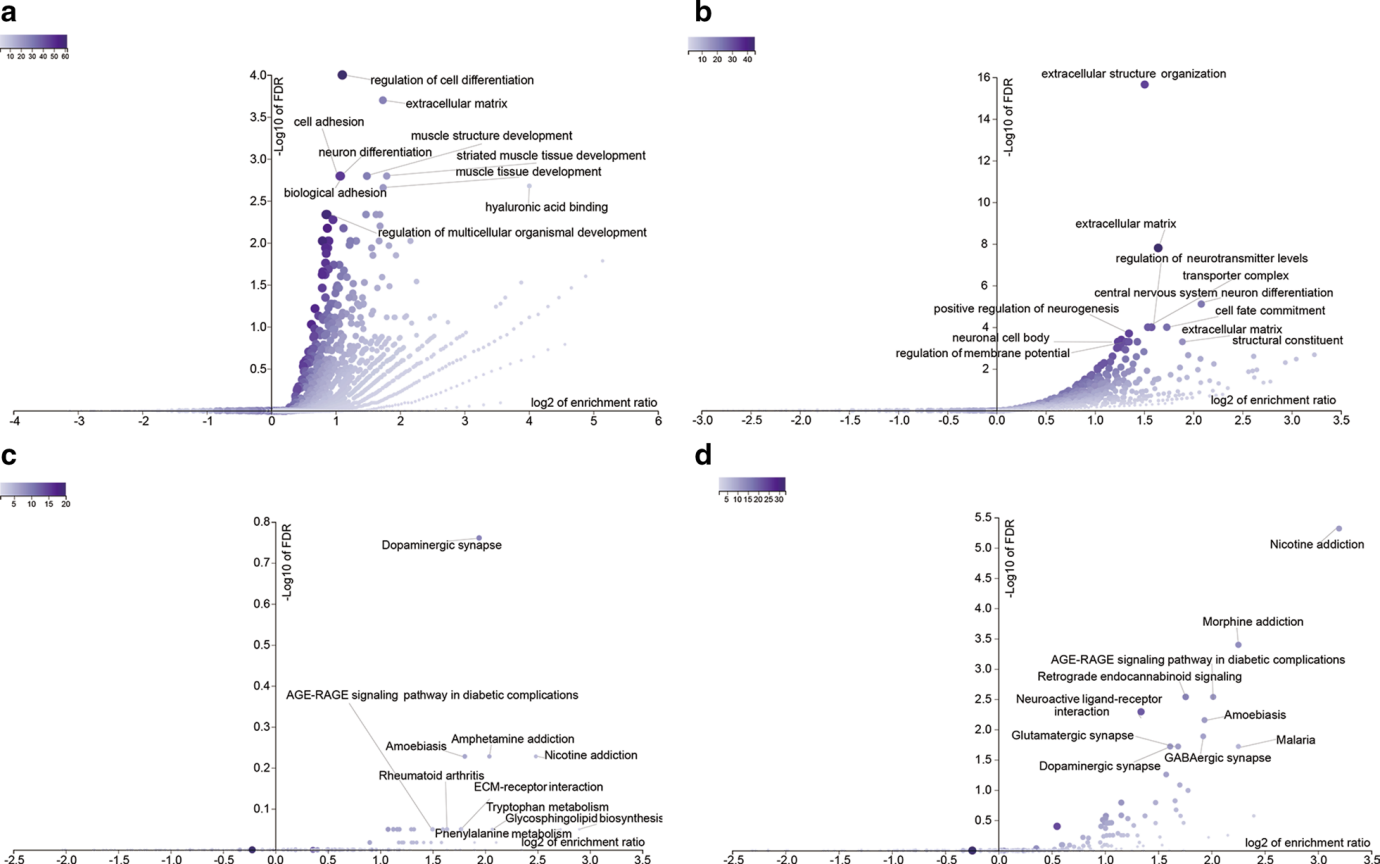
Results of enriched GO terms and pathways in GSE112047 and GSE76938 datasets. Representative enriched GO terms in GSE112047 (**a**) and GSE76938 (**b**) datasets and representative pathways in GSE112047 (**c**) and GSE76938 (**d**) datasets

GO term enrichment analysis was perform based on DNA methylation-driven genes obtained from GEO database. The results of GO analyses demonstrated that DNA methylation-driven genes in GSE112047 were mainly enriched in regulation of cell differentiation, extracellular matrix, cell adhesion, muscle structure development, and neuron differentiation (Fig. 12a and Table 5). DNA methylation-driven genes in GSE76938 were mainly enriched in extracellular structure organization, extracellular matrix, central nervous system neuron differentiation, regulation of neurotransmitter levels, and cell fate commitment (Fig. 12b and Table 5).

**Table 5:** GO terms enriched in GSE112047 and GSE76938 datasets

Gene set	Description	Size	Expect	Ratio	p value	FDR
GSE112047						
GO:0045595	Regulation of cell differentiation	1699	28.866	2.1479	5.48E−09	0.0001
GO:0031012	Extracellular matrix	496	8.4271	3.3226	3.00E−08	0.0002
GO:0007155	Cell adhesion	1369	23.259	2.1067	5.21E−07	0.0016
GO:0061061	Muscle structure development	610	10.364	2.7982	6.40E−07	0.0016
GO:0030182	Neuron differentiation	1313	22.308	2.1069	9.19E−07	0.0016
GO:0005540	Hyaluronic acid binding	22	0.3738	16.052	1.3596E−06	0.0021
GO:2000026	Regulation of multicellular organismal development	1908	32.417	1.82	3.8179E−06	0.0046
GSE76938						
GO:0043062	Extracellular structure organization	400	11.623	2.8392	7.43E−08	0
GO:0031012	Extracellular matrix	496	14.412	3.1223	1.20E−11	1.56E−08
GO:0021953	Central nervous system neuron differentiation	179	5.2012	4.2298	1.19E−08	7.73E−06
GO:0001505	Regulation of neurotransmitter levels	335	9.7341	2.9792	1.70E−07	0.0001
GO:0045165	Cell fate commitment	249	7.2352	3.3171	2.86E−07	0.0001
GO:1990351	Transporter complex	332	9.6469	2.9025	4.73E−07	0.0001
GO:0050769	Positive regulation of neurogenesis	447	12.989	2.5407	9.50E−07	0.0002
GO:0043025	Neuronal cell body	486	14.122	2.4076	2.17E−06	0.0004
GO:0005201	Extracellular matrix structural constituent	158	4.591	3.7029	3.55E−06	0.0005
GO:0042391	Regulation of membrane potential	414	12.03	2.4938	4.34E−06	0.0005

### KEGG pathway analysis of DNA methylation-driven genes obtained from GEO

KEGG enrichment analysis was perform based on DNA methylation-driven genes obtained from GEO database. The results of KEGG analyses demonstrated that DNA methylation-driven genes in GSE112047 were mainly enriched in Dopaminergic synapse, Nicotine addiction, and Amphetamine addiction pathways (Fig. 12c and Table 6). DNA methylation-driven genes in GSE76938 were mainly enriched in Nicotine addiction, Morphine addiction, and Retrograde endocannabinoid signaling pathways (Fig. 12d and Table 6).

**Table 6:** Pathways enriched in GSE112047 and GSE76938 datasets

Gene set	Description	Size	Expect	Ratio	p value	FDR
GSE112047						
hsa04728	Dopaminergic synapse	131	2.3422	3.8426	0.0005	0.1734
hsa05033	Nicotine addiction	40	0.7152	5.5931	0.0054	0.5917
hsa05031	Amphetamine addiction	68	1.2158	4.1126	0.0072	0.5917
hsa05146	Amoebiasis	96	1.7164	3.4957	0.0073	0.5917
hsa04512	ECM-receptor interaction	82	1.4661	3.4104	0.0154	0.8913
hsa05323	Rheumatoid arthritis	90	1.6091	3.1073	0.0222	0.8913
hsa00604	Glycosphingolipid biosynthesis	15	0.2682	7.4574	0.0286	0.8913
hsa04933	AGE-RAGE signaling pathway in diabetic complications	99	1.77	2.8248	0.0318	0.8913
hsa00380	Tryptophan metabolism	40	0.7152	4.1948	0.0341	0.8913
hsa00360	Phenylalanine metabolism	17	0.3039	6.5801	0.0362	0.8913
GSE76938						
hsa05033	Nicotine addiction	40	1.1993	9.1721	1.47E−08	4.8E−06
hsa05032	Morphine addiction	91	2.7284	4.7647	2.68E−06	0.0004
hsa04723	Retrograde endocannabinoid signaling	148	4.4374	3.3804	0	0.0029
hsa04933	AGE-RAGE signaling pathway in diabetic complications	99	2.9682	4.0428	0	0.0029
hsa04080	Neuroactive ligand-receptor interaction	277	8.305	2.5286	0.0001	0.0051
hsa05146	Amoebiasis	96	2.8783	3.8217	0.0001	0.007
hsa04727	GABAergic synapse	88	2.6384	3.7901	0.0003	0.013
hsa04728	Dopaminergic synapse	131	3.9277	3.0553	0.0005	0.0191
hsa05144	Malaria	49	1.4691	4.7647	0.0006	0.0191
hsa04724	Glutamatergic synapse	114	3.418	3.2183	0.0006	0.0191

### Validation of MAOB and RTP4 expression in the Human Protein Atlas and TCGA

**Fig. 13:**
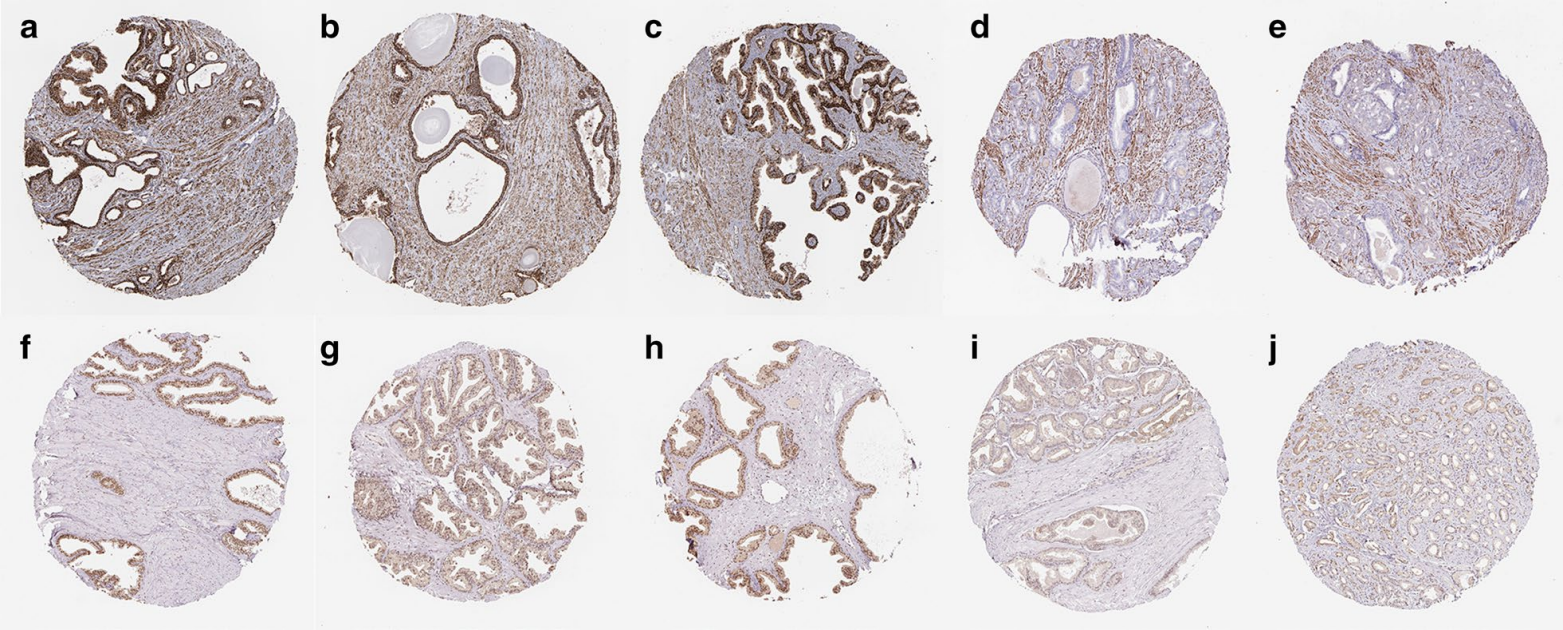
Validation of MAOB and RTP4 expression in the Human Protein Atlas. With respect to MAOB, when compared with normal prostate tissues (**a**–**c**), the results demonstrated that the staining was not detected and the intensity was negative in low grade (**d**) and high grade (**e**) prostate adenocarcinoma, respectively. Also, in terms of RTP4, when compared with normal prostate tissues (**f**–**h**), the results demonstrated that the staining was low and the intensity was weak in low grade (**i**) and high grade (**j**) prostate adenocarcinoma

The results of stains on normal prostate tissues demonstrated that the MAOB (Fig. 13a–c, patient id 1938, 2053, and 2098) and RTP4 were highly expressed in normal prostate tissues. With respect to MAOB, the staining was not detected and the intensity was negative in low grade prostate (Patient id 3910) adenocarcinoma (Fig. 13d). Also, the staining was not detected and the intensity was negative in high grade prostate (Patient id 3561) adenocarcinoma (Fig. 13e).

**Fig. 14:**
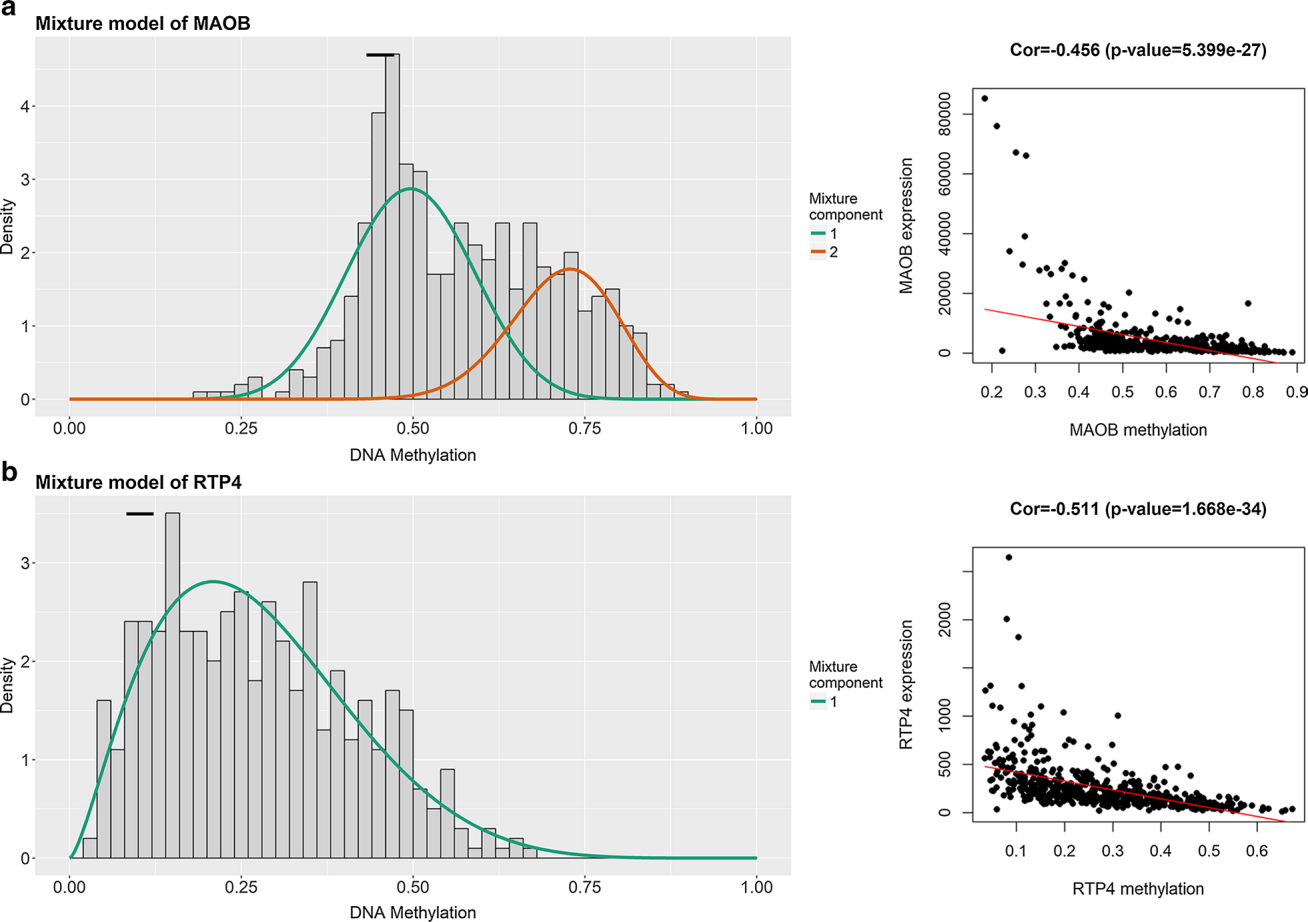
Methylation status and correlation between genes expression and DNA methylation of MAOB (**a**) and RTP4 (**b**). Average β-values are presented on the x-axis, log2 FPKM gene expression values are presented on y-axis

Plus, the results of stains on normal prostate tissues demonstrated that the RTP4 (Fig. 13f–h, patient id 1798, 1984, and 3497) were highly expressed in normal prostate tissues. In terms of RTP4, the staining was low and the intensity was weak in low grade prostate (Patient id 4525) adenocarcinoma (Fig. 13i). The staining was low and the intensity was weak in high grade prostate (Patient id 4347) adenocarcinoma (Fig. 13j). Methylation status and correlation between genes expression and DNA methylation of MAOB and RTP4 were demonstrated in Fig. 14a, b.

## Discussion

Epigenetic changes and modifications are a crucial component of initiation and progression of tumorigenesis [14]. DNA methylation has been most studied and hypermethylaiton is associated with a silencing effect on tumor suppressor genes. Previous studies [15–17] reported that the modification of the methylation status of specific genes has been associated with worse prognosis, which demonstrated that the modification of epigenetics may be involved in the progression of tumorigenesis.

Previous studies reveals that DNA methylation plays an important role in the development of prostate cancer and associates with adverse clinical outcomes. The methylation state of promoters of specific genes were also involved in the development of prostate cancer [11, 18, 19]. Larsen et al. [20] reported that DNA-methylation analysis of urine cells captured by microfiltration provides a novel tool for noninvasive detection of high-grade prostate cancer.

The novel genes involved in the epigenetic regulation of prostate cancer could be identified by using high-throughput arrays [7, 9, 18]. We aimed to elucidate the role and importance of DNA methylation in prostate cancer by analyzing The Cancer Genome Atlas Project. In this study, a model-based tool (MethyMix) was used to identify key genes with aberrant methylation and gene expression was linked to aberrant methylation. In this study, the top 3 hypermethylated genes were demonstrated including HIST1H2BH, FAM200A, and ZFP36L2 and the top 3 hypomethylated genes were also demonstrated including TAF1D, TMEM87A, and KLK2.

The GO term analysis revealed that the differentially expressed DNA methylation driven genes were involved in oxidation–reduction process, extracellular exosome, electron carrier activity, response to reactive oxygen species, and aldehyde dehydrogenase [NAD(P)+] activity. Furthermore, the enriched KEGG pathway of the differentially expressed DNA methylation driven genes including drug metabolism—cytochrome P450, phenylalanine metabolism, histidine metabolism, glutathione metabolism, and tyrosine metabolism. PPI analysis demonstrated that hub genes were IFITM1, RTP4, ACSF2, GSTM2, GSTM1, ACOX2, COL4A6, ITGA2, AKR1B1, NPY, CFTR, GPX7, ALDH3A1, CRYZ, ALDH2, MAOB, GSTP1, GPX3, XAF1, and BST2, which might play important roles in DNA methylation in prostate cancer patients. We then overlapped the differentially methylated genes and identified a common list of 6 methylated genes including AKR1B1, RTP4, MAOB, GSTM2, GPX3, and COL4A6.

In terms of GSTM2, Angulo et al. [21] revealed that GSTM2 hypermethylation could be used to predict biochemical recurrence after radical prostatectomy, which suggested that epigenetic silencing of GSTM2 played an important role in involving biochemical recurrence. Plus, Ashour et al. [22] demonstrated that GSTM2 was hypermethylated in PCa and was simultaneously methylated in 40.9% if the PCa, which revealed that epigenetic silencing of GSTM2 is a common event in PCa. Maldonado et al. [23] revealed that GPX3 was aberrantly methylated and silenced in PCa tissues and had tumor suppressor activity in PCa cell lines. Plus, Lin et al. [24] revealed that GPX3, as a DNA methylation-silenced tumor suppressor gene, was hypermethylated in PCa. Strand et al. [25] stated that hypermethylation of COL4A6 could be treated as a frequent target in PCa. Ikeda et al. [26] demonstrated that hypermethylation of COL4A5 was one of the events that was responsible for the development of colorectal cancer.

With respect to AKR1B1, Theresa et al. [27] stated that AKR1B1 promoter methylation proved to be commonly methylated in breast cancer. Also, Wei et al. [28] demonstrated that AKR1B1 could be potential screening markers of colorectal carcinoma. However, the relationship between AKR1B1 methylation and PCa has not been elucidated yet. To the best of our knowledge, RTP4 and MAOB have not been reported to be associated with DNA methylation in the occurrence and development of cancer, which means RTP4 and MAOB could be regarded as potential targets for the new therapeutic managements in prostate cancer. The Human Protein Atlas validated the staining and intensity of RTP4 and MAOB in PCa tissue, the results were consistent with bioinformatic analysis that MAOB and RTP4 may be novel biomarkers in PCa.

In this study, GO term enrichment analysis showed that extracellular exosome was associated with DNA methylation in prostate cancer. Huang et al. [29] demonstrated that extracellular exosomes, especially plasma exosomes, containing miR-1290 and miR-375 may serves as promising prognostic biomarkers for castration-resistant prostate cancer (CRPC). Bryzgunova et al. [30] also demonstrated that extracellular vesicles founded from urine showed a high specificity and sensitivity in distinguishing prostate cancer patients from healthy donors. GO term enrichment analysis also revealed that oxidation–reduction process was also involved in DNA methylation of prostate cancer. Schlaepfer et al. [31] revealed that inhibition of lipid oxidation plays a crucial role in elevating glucose metabolism of PCa cells. Liu et al. [32] also demonstrated that oxidation–reduction reactions was associated with mitochondria and mitochondrial damage in DU145 and PC3 prostate cancer cell lines.

In this study, KEGG pathway enrichment analysis showed that cytochrome P450 pathway was involved in the methylation process in PCa. Chen et al. [33] demonstrated that cytochrome P450 (CYP) enzymes including CYP2R1, CYP27B1 and CYP24A1 were involved in the development and progression of PCa and were treated as promising targets in the treatment of PCa. Mandić et al. [34] also revealed that CYP1A1 can bind to DNA and induced the carcinogenesis of prostate cancer via involving in the various endogenous and environmental reactive compounds. The phenylalanine metabolism was also found to be participant in the methylation process in PCa. Gueron et al. [35] reported that the concomitant resistance (CR) phenomenon occurs in murine prostate cancer accompanied with ROS-damaged phenylalanine and the CR phenomenon was reversed when the phenylalanine were injected into mice. Lapek et al. demonstrated that the histidine-phosphorylated proteins with diverse functions including metabolism, protein folding, and motility play important roles in the development and progression of PCa.

## Conclusion

Methylated hub genes, including MAOB and RTP4, can be regarded as novel biomarkers for accurate PCa diagnosis and treatment. Further studies are needed to draw more attention to the roles of these hub genes in the occurrence and development of PCa.

## Methods

### Data source

Expressing profiles of gene-specific DNA methylation data and clinical information of patients with PCa were downloaded from The Cancer Genome Atlas (TCGA) database (https://tcga-data.nci.nih.gov/tcga/) [36]. The clinicopathological information of the patients with prostate cancer were also extracted. Illumina Human Methylation 450 Beadchip (450 K array) was used to measure the DNA methylation data. A total of 482,421 CpG sites throughout the genome would be assessed [37]. We downloaded level 3 methylation data and level 3 RNA-seq data from the TCGA data portal by using the TCGA-Assembler [38]. Two gene methylation datasets (GSE112047 and GSE76938) were downloaded from the Gene Expression Omnibus (GEO, https://www.ncbi.nlm.nih.gov/geo/). The gene methylation microarray datasets were composed of 16 and 63 normal prostate tissues, respectively, and 16 and 73 PCa tumor tissues, respectively (GPL13534 Illumina HumanMethylation450 BeadChip).

### Identification of DNA methylation-driven genes

The expression and methylation were analyzed with R 3.4.4 software (https://www.r-project.org/). For the data from TCGA, the R package MethylMix was applied to perform an analysis integrating gene expression data and methylation data. MethylMix was designed to identify gene expression that were correlated with methylation events. A total of three parts of MethylMix analysis were described as previous studies [39, 40]. For the data from GEO, the R package limma was applied to perform analysis to identify the DMEs. Also, VennDiagram package in R software was used to identify overlapping DNA methylation-driven genes.

### Gene ontology analysis

Gene ontology analysis (GO) (http://david.abcc.ncifcrf.gov/) is used for annotating differentially methylated DNA methylation-driven genes [41]. The Database for Annotation, Visualization, and Integrated Discovery (DAVID) (http://david.abcc.ncifcrf.gov/) tool was used for obtaining the enriched GO terms of differentially methylated DNA methylation-driven genes based on the hypergeometric distribution to compute values, which was described as previous study [42]. FDR < 0.05 was set as the threshold value.

### Pathway analysis

The gene lists found to be statistically significant by MethylMix were used to perform pathway analysis. The Kyoto Encyclopedia of Genes and Genomes (KEGG; http://www.kegg.jp/) was used to perform the pathway enrichment analysis. Furthermore, the ConsensusPathDB was also used to validate the enriched pathway results. ConsensusPathDB was used to performed pathway analysis as previous studies described [40, 43]. The default settings were as follow: minimum overlap and p value cutoff of 0.01.

### Integration of protein–protein interaction (PPI) network

Search Tool for the Retrieval of Interacting Genes (STRING) database (version 10.5) was used to evaluated the PPI information. Differentially methylated DNA methylation-driven genes were mapped to STRING to evaluate the interactive relationships of these genes. Cytoscape software (version 3.6.1) was used to constructed the PPI networks.

### Validation of the candidate hub genes screened from TCGA in GEO database and the Human Protein Atlas

To confirm the candidate hub genes we obtained, two datasets (GSE76938 and GSE112047) was downloaded from GEO database to validate the methylation levels of these candidate hub genes. To confirm the expression of these hub genes in PCa tissues, immunohistochemistry of these hub genes were evaluated by the Human Protein Atlas (http://www.proteinatlas.org). The expressions of MAOB and RTP4 were validated in The Human Protein Atlas.

## Supplementary information

**Additional file 1: Table S1.** A gene list of 266 DNA methylation driven genes in TCGA.

**Additional file 2: Table S2.** A gene list of 369 differentially methylated genes and 594 differentially methylated genes in GSE112047 and GSE76938.

## Data Availability

All data was obtained from The Cancer Genome Atlas (TCGA, https://tcga-data.nci.nih.gov/tcga/) and Gene Expression Omnibus (GEO, https://www.ncbi.nlm.nih.gov/geo/).

## Abbreviations

TCGA: The Cancer Genome Atlas
GEO: Gene Expression Omnibus
KEGG: Kyoto Encyclopedia of Genes and Genomes Pathway
PPI: the protein–protein interaction
PCa: prostate cancer
DAVID: The Database for Annotation, Visualization, and Integrated Discovery
STRING: Search Tool for the Retrieval of Interacting Genes Database
CRPC: castration-resistant prostate cancer
CYP: cytochrome P450
CR: concomitant resistance
