# Supplementary material for: Identification of key DNA methylation-driven genes in prostate adenocarcinoma: an integrative analysis of TCGA methylation data
Source: J Transl Med. 2019 Sep 18;17:311. doi: 10.1186/s12967-019-2065-2 (PMC6751626; doi:10.1186/s12967-019-2065-2)
Supplement: Supplementary file 1 — Additional file 1: Table S1. A gene list of 266 DNA methylation driven genes in TCGA. [file 12967_2019_2065_MOESM1_ESM.docx]

| Table S1. A gene list of 266 DNA methylation driven genes in TCGA. | | | | | | | |
| --- | --- | --- | --- | --- | --- | --- | --- |
| gene | normalMean | TumorMean | logFC | pValue | adjustP | cor | corPavlue |
| LRP4 | 0.214477122 | 0.326813308 | 0.607642963 | 6.47E-26 | 2.04E-23 | -0.416294888 | 2.46E-22 |
| RND2 | 0.277652066 | 0.404620032 | 0.543289612 | 6.70E-26 | 2.11E-23 | -0.394536318 | 4.93E-20 |
| SLC16A5 | 0.239520886 | 0.387805788 | 0.695182873 | 9.38E-26 | 2.95E-23 | -0.654331161 | 2.64E-62 |
| B3GNT8 | 0.146887514 | 0.282685687 | 0.944487072 | 1.17E-25 | 3.69E-23 | -0.599863697 | 4.34E-50 |
| COL4A6 | 0.39367086 | 0.635961278 | 0.691949001 | 1.96E-25 | 6.16E-23 | -0.677984063 | 1.94E-68 |
| PLA2G3 | 0.415164953 | 0.561044781 | 0.434431265 | 2.29E-25 | 7.21E-23 | -0.404801124 | 4.24E-21 |
| KLK2 | 0.42230197 | 0.252663444 | -0.741058027 | 2.34E-25 | 7.36E-23 | -0.424469057 | 3.03E-23 |
| AS3MT | 0.139322623 | 0.198548425 | 0.511061377 | 2.51E-25 | 7.92E-23 | -0.35064352 | 6.98E-16 |
| HIF3A | 0.35147053 | 0.560561229 | 0.673468236 | 2.77E-25 | 8.73E-23 | -0.430543536 | 6.16E-24 |
| LAYN | 0.22335296 | 0.392433566 | 0.813123071 | 3.74E-25 | 1.18E-22 | -0.523245102 | 2.03E-36 |
| RPS2 | 0.660016277 | 0.47194894 | -0.483870821 | 4.41E-25 | 1.39E-22 | -0.396809746 | 2.88E-20 |
| GPX7 | 0.286025677 | 0.456385602 | 0.674108612 | 4.86E-25 | 1.53E-22 | -0.743428117 | 7.23E-89 |
| APOBEC3C | 0.165693324 | 0.279582247 | 0.75475728 | 5.47E-25 | 1.72E-22 | -0.6653404 | 4.33E-65 |
| AOX1 | 0.292763673 | 0.523540773 | 0.838565346 | 5.71E-25 | 1.80E-22 | -0.604125362 | 5.83E-51 |
| FAXDC2 | 0.267491887 | 0.45579696 | 0.768896165 | 6.09E-25 | 1.92E-22 | -0.758231788 | 2.23E-94 |
| PYCARD | 0.189564988 | 0.353218395 | 0.897867948 | 6.58E-25 | 2.07E-22 | -0.460232967 | 1.58E-27 |
| ARL4C | 0.442964367 | 0.554917063 | 0.325081514 | 6.77E-25 | 2.13E-22 | -0.548038424 | 1.85E-40 |
| GSTP1 | 0.159638122 | 0.275893224 | 0.78930481 | 8.19E-25 | 2.58E-22 | -0.587888986 | 1.04E-47 |
| CCK | 0.278860811 | 0.447750654 | 0.683150335 | 1.02E-24 | 3.22E-22 | -0.366841879 | 2.43E-17 |
| DUOX1 | 0.422970478 | 0.5334638 | 0.334833406 | 1.30E-24 | 4.11E-22 | -0.370752595 | 1.05E-17 |
| EFS | 0.214080765 | 0.482979034 | 1.173805388 | 1.37E-24 | 4.31E-22 | -0.679784617 | 6.28E-69 |
| KLF8 | 0.384117077 | 0.694360124 | 0.854137993 | 1.49E-24 | 4.68E-22 | -0.609174931 | 5.20E-52 |
| CAPG | 0.350294368 | 0.472296815 | 0.431126013 | 1.58E-24 | 4.98E-22 | -0.561610962 | 8.08E-43 |
| NYNRIN | 0.283798134 | 0.396190291 | 0.481328424 | 1.60E-24 | 5.05E-22 | -0.591806886 | 1.78E-48 |
| SOSTDC1 | 0.329497475 | 0.470754699 | 0.514708087 | 1.71E-24 | 5.38E-22 | -0.412203699 | 6.87E-22 |
| RARB | 0.442643002 | 0.593414974 | 0.42289772 | 1.87E-24 | 5.90E-22 | -0.547504289 | 2.27E-40 |
| ELF4 | 0.287374124 | 0.426851292 | 0.570803384 | 1.87E-24 | 5.90E-22 | -0.600868494 | 2.71E-50 |
| CDC42EP5 | 0.307235867 | 0.424949179 | 0.467943666 | 1.89E-24 | 5.95E-22 | -0.391917463 | 9.08E-20 |
| KLK1 | 0.56369274 | 0.691820277 | 0.295488311 | 1.92E-24 | 6.04E-22 | -0.360295106 | 9.66E-17 |
| MARVELD1 | 0.233511159 | 0.411679817 | 0.818031228 | 2.03E-24 | 6.40E-22 | -0.614956168 | 3.09E-53 |
| HAPLN3 | 0.429211387 | 0.530547769 | 0.305794301 | 2.10E-24 | 6.62E-22 | -0.338651232 | 7.43E-15 |
| PROM1 | 0.420577646 | 0.562655664 | 0.419880115 | 2.45E-24 | 7.72E-22 | -0.397764763 | 2.30E-20 |
| WFDC2 | 0.125845936 | 0.347071667 | 1.463574969 | 2.89E-24 | 9.09E-22 | -0.468741891 | 1.27E-28 |
| TMEM106A | 0.229277616 | 0.535666436 | 1.22423939 | 3.00E-24 | 9.44E-22 | -0.679887951 | 5.89E-69 |
| HLF | 0.227068543 | 0.377160317 | 0.732050037 | 3.18E-24 | 1.00E-21 | -0.568966503 | 3.83E-44 |
| CX3CL1 | 0.554953992 | 0.656899185 | 0.243303804 | 3.36E-24 | 1.06E-21 | -0.5251029 | 1.04E-36 |
| CCDC8 | 0.330584534 | 0.533370541 | 0.690118912 | 3.79E-24 | 1.19E-21 | -0.664004768 | 9.57E-65 |
| C8orf88 | 0.222617683 | 0.402364 | 0.853933038 | 3.98E-24 | 1.25E-21 | -0.564276675 | 2.70E-43 |
| CBLL1 | 0.850164111 | 0.715650008 | -0.248487155 | 4.35E-24 | 1.37E-21 | -0.377426459 | 2.44E-18 |
| RNF152 | 0.206028915 | 0.390427056 | 0.922206208 | 4.57E-24 | 1.44E-21 | -0.518813023 | 9.89E-36 |
| EOGT | 0.275053567 | 0.415657187 | 0.595681543 | 6.66E-24 | 2.10E-21 | -0.563045615 | 4.48E-43 |
| AKR1B1 | 0.298906304 | 0.500418305 | 0.743441239 | 7.32E-24 | 2.31E-21 | -0.498577616 | 1.02E-32 |
| MFAP4 | 0.404893596 | 0.589570447 | 0.542121385 | 7.43E-24 | 2.34E-21 | -0.383647211 | 6.05E-19 |
| CCDC181 | 0.336348481 | 0.576071294 | 0.776290626 | 8.28E-24 | 2.61E-21 | -0.394742869 | 4.69E-20 |
| SYNGR2 | 0.444855164 | 0.323098816 | -0.461360237 | 8.77E-24 | 2.76E-21 | -0.461510414 | 1.09E-27 |
| ACOX2 | 0.479316486 | 0.599785155 | 0.323467254 | 9.02E-24 | 2.84E-21 | -0.693681274 | 7.77E-73 |
| GSTM2 | 0.17438679 | 0.346586657 | 0.990925358 | 9.69E-24 | 3.05E-21 | -0.58439819 | 4.93E-47 |
| FBXO17 | 0.118028402 | 0.282400386 | 1.258607996 | 9.97E-24 | 3.14E-21 | -0.679568506 | 7.20E-69 |
| TTC28 | 0.40963399 | 0.54897709 | 0.422410514 | 1.01E-23 | 3.17E-21 | -0.434441869 | 2.18E-24 |
| RARRES2 | 0.571691299 | 0.681604172 | 0.253697834 | 1.02E-23 | 3.22E-21 | -0.354689329 | 3.07E-16 |
| FAIM2 | 0.310979992 | 0.475877701 | 0.613769088 | 1.05E-23 | 3.29E-21 | -0.361370371 | 7.72E-17 |
| RAB34 | 0.233180203 | 0.382772135 | 0.715040501 | 1.10E-23 | 3.47E-21 | -0.543840916 | 9.42E-40 |
| TRIP6 | 0.603273567 | 0.737157752 | 0.289161019 | 1.25E-23 | 3.94E-21 | -0.466024475 | 2.87E-28 |
| SLC39A7 | 0.737439878 | 0.55783243 | -0.402693626 | 1.36E-23 | 4.27E-21 | -0.400049276 | 1.33E-20 |
| SLC16A2 | 0.179356472 | 0.33077461 | 0.883018693 | 1.53E-23 | 4.81E-21 | -0.648013779 | 9.27E-61 |
| CYBA | 0.37125419 | 0.576567492 | 0.63508219 | 1.56E-23 | 4.90E-21 | -0.432608254 | 3.56E-24 |
| ANGPTL1 | 0.664966537 | 0.806478883 | 0.278355015 | 1.66E-23 | 5.23E-21 | -0.416144485 | 2.55E-22 |
| GRASP | 0.483612189 | 0.61432129 | 0.345142776 | 2.09E-23 | 6.59E-21 | -0.441241665 | 3.44E-25 |
| GRIA3 | 0.314513262 | 0.503301085 | 0.678300854 | 2.12E-23 | 6.69E-21 | -0.454908458 | 7.41E-27 |
| ANXA2 | 0.293801299 | 0.434032982 | 0.5629639 | 2.19E-23 | 6.91E-21 | -0.648094182 | 8.87E-61 |
| ACSF2 | 0.410563277 | 0.559539219 | 0.446634664 | 2.27E-23 | 7.14E-21 | -0.547692822 | 2.11E-40 |
| RAPGEFL1 | 0.222359223 | 0.346102015 | 0.638305094 | 2.29E-23 | 7.21E-21 | -0.390194295 | 1.35E-19 |
| GPRASP1 | 0.237104644 | 0.491673619 | 1.052177027 | 2.33E-23 | 7.35E-21 | -0.57732355 | 1.09E-45 |
| PLBD1 | 0.221778714 | 0.379907412 | 0.776526957 | 2.33E-23 | 7.35E-21 | -0.551839955 | 4.13E-41 |
| RPL13A | 0.39770426 | 0.29194025 | -0.446022886 | 3.01E-23 | 9.47E-21 | -0.317163579 | 4.01E-13 |
| DNAJA3 | 0.590109326 | 0.478111819 | -0.303634189 | 3.30E-23 | 1.04E-20 | -0.394799867 | 4.63E-20 |
| C2orf88 | 0.401988714 | 0.537012526 | 0.417800742 | 3.46E-23 | 1.09E-20 | -0.579990132 | 3.42E-46 |
| RGN | 0.505969612 | 0.697881535 | 0.463931421 | 3.61E-23 | 1.14E-20 | -0.650353225 | 2.51E-61 |
| PON3 | 0.340338746 | 0.524332765 | 0.623511297 | 3.68E-23 | 1.16E-20 | -0.502113996 | 3.15E-33 |
| NEFH | 0.239325981 | 0.376835186 | 0.654956655 | 3.75E-23 | 1.18E-20 | -0.462705476 | 7.66E-28 |
| ANKDD1B | 0.221957553 | 0.443551592 | 0.998818119 | 4.06E-23 | 1.28E-20 | -0.474461264 | 2.24E-29 |
| SLC43A3 | 0.192533906 | 0.333860465 | 0.794132725 | 4.09E-23 | 1.29E-20 | -0.414308845 | 4.06E-22 |
| RIPPLY2 | 0.061609773 | 0.166659059 | 1.43566861 | 4.35E-23 | 1.37E-20 | -0.332153018 | 2.57E-14 |
| TMLHE | 0.263311977 | 0.437034022 | 0.730972449 | 4.43E-23 | 1.40E-20 | -0.643938975 | 8.81E-60 |
| ITPRIPL1 | 0.317980898 | 0.488528583 | 0.619502873 | 6.00E-23 | 1.89E-20 | -0.653442607 | 4.38E-62 |
| MOB3B | 0.305779656 | 0.432906144 | 0.501561851 | 6.41E-23 | 2.02E-20 | -0.350421098 | 7.30E-16 |
| LTF | 0.321502299 | 0.487299887 | 0.599980836 | 6.77E-23 | 2.13E-20 | -0.341716592 | 4.10E-15 |
| FBXO30 | 0.177043658 | 0.281051814 | 0.666730958 | 8.55E-23 | 2.69E-20 | -0.382297401 | 8.21E-19 |
| COL7A1 | 0.498300526 | 0.638600544 | 0.357897685 | 9.55E-23 | 3.01E-20 | -0.32436841 | 1.09E-13 |
| EPHX3 | 0.247318651 | 0.465247991 | 0.911628882 | 9.78E-23 | 3.08E-20 | -0.435526721 | 1.63E-24 |
| DSC3 | 0.15714371 | 0.282654433 | 0.846954797 | 1.03E-22 | 3.26E-20 | -0.34822378 | 1.13E-15 |
| KLK10 | 0.132655021 | 0.233389496 | 0.815060349 | 1.08E-22 | 3.39E-20 | -0.345132456 | 2.10E-15 |
| HOXD11 | 0.174447669 | 0.331033159 | 0.92418142 | 1.46E-22 | 4.58E-20 | -0.591704909 | 1.86E-48 |
| IVNS1ABP | 0.422123353 | 0.560254515 | 0.408417724 | 1.72E-22 | 5.41E-20 | -0.371324268 | 9.27E-18 |
| TP53AIP1 | 0.595460574 | 0.708849984 | 0.251474347 | 1.93E-22 | 6.07E-20 | -0.545581269 | 4.81E-40 |
| CANT1 | 0.477686117 | 0.346059864 | -0.465041321 | 1.97E-22 | 6.22E-20 | -0.576416936 | 1.61E-45 |
| HAAO | 0.212661296 | 0.420342476 | 0.983007762 | 2.19E-22 | 6.91E-20 | -0.523662788 | 1.75E-36 |
| S100A16 | 0.619856005 | 0.736397497 | 0.248551612 | 2.23E-22 | 7.04E-20 | -0.539677408 | 4.64E-39 |
| KLK3 | 0.528659515 | 0.348234132 | -0.602281232 | 2.38E-22 | 7.50E-20 | -0.352378214 | 4.92E-16 |
| TMEM87A | 0.36898334 | 0.219989007 | -0.746124244 | 2.40E-22 | 7.57E-20 | -0.415783216 | 2.80E-22 |
| ANKRD65 | 0.506626049 | 0.632878317 | 0.321006882 | 2.48E-22 | 7.82E-20 | -0.595797752 | 2.86E-49 |
| USP44 | 0.385291773 | 0.498152781 | 0.370636897 | 2.77E-22 | 8.73E-20 | -0.450739848 | 2.43E-26 |
| GPR62 | 0.316303425 | 0.419666108 | 0.407932779 | 2.86E-22 | 9.02E-20 | -0.319374118 | 2.70E-13 |
| UBXN10 | 0.512869526 | 0.633121832 | 0.303891295 | 3.24E-22 | 1.02E-19 | -0.514657095 | 4.27E-35 |
| MPC2 | 0.847716806 | 0.573604393 | -0.563526315 | 3.24E-22 | 1.02E-19 | -0.509873152 | 2.25E-34 |
| EEF2 | 0.462781275 | 0.341445622 | -0.438674653 | 3.47E-22 | 1.09E-19 | -0.565145786 | 1.88E-43 |
| TAF1D | 0.298851771 | 0.17046481 | -0.809956148 | 5.12E-22 | 1.61E-19 | -0.515133904 | 3.62E-35 |
| KRT7 | 0.433389723 | 0.585741783 | 0.434599868 | 5.48E-22 | 1.73E-19 | -0.557550597 | 4.21E-42 |
| DES | 0.455944276 | 0.568095764 | 0.317276631 | 8.55E-22 | 2.69E-19 | -0.354203698 | 3.39E-16 |
| TAGLN | 0.267337688 | 0.405013775 | 0.599307739 | 8.71E-22 | 2.74E-19 | -0.413370004 | 5.13E-22 |
| GPX3 | 0.321642722 | 0.453864398 | 0.496802281 | 8.95E-22 | 2.82E-19 | -0.433028182 | 3.18E-24 |
| SPATA6 | 0.236108364 | 0.355022698 | 0.588462112 | 9.97E-22 | 3.14E-19 | -0.566416242 | 1.11E-43 |
| CLIP4 | 0.279235845 | 0.39463813 | 0.499046203 | 1.22E-21 | 3.85E-19 | -0.594112278 | 6.21E-49 |
| ITGA2 | 0.296951434 | 0.420262564 | 0.501063951 | 1.68E-21 | 5.28E-19 | -0.515097091 | 3.66E-35 |
| PMEPA1 | 0.427234317 | 0.301507194 | -0.50283511 | 1.93E-21 | 6.07E-19 | -0.559861766 | 1.65E-42 |
| SLFN11 | 0.125013269 | 0.281974904 | 1.173485537 | 2.02E-21 | 6.38E-19 | -0.367762704 | 2.00E-17 |
| TAL1 | 0.245289423 | 0.381632926 | 0.63770062 | 2.52E-21 | 7.94E-19 | -0.370079185 | 1.21E-17 |
| LGI1 | 0.568854716 | 0.68266068 | 0.26310842 | 2.95E-21 | 9.29E-19 | -0.378024357 | 2.13E-18 |
| CSTA | 0.336435934 | 0.54657901 | 0.700098252 | 3.02E-21 | 9.50E-19 | -0.334234232 | 1.73E-14 |
| AMT | 0.471485011 | 0.60709109 | 0.36470109 | 3.18E-21 | 1.00E-18 | -0.683545204 | 5.78E-70 |
| STAP1 | 0.437790174 | 0.338432073 | -0.371373274 | 3.93E-21 | 1.24E-18 | -0.377533411 | 2.38E-18 |
| SERPINB1 | 0.214641844 | 0.40421979 | 0.913208603 | 4.24E-21 | 1.33E-18 | -0.675856255 | 7.31E-68 |
| PAH | 0.193428244 | 0.327209999 | 0.758418367 | 4.29E-21 | 1.35E-18 | -0.335422616 | 1.38E-14 |
| ARL9 | 0.309907877 | 0.517193011 | 0.738863355 | 4.47E-21 | 1.41E-18 | -0.379876184 | 1.41E-18 |
| FASN | 0.639005053 | 0.492819683 | -0.374767463 | 4.63E-21 | 1.46E-18 | -0.579493565 | 4.25E-46 |
| RPL7A | 0.769000171 | 0.599858625 | -0.358361393 | 5.15E-21 | 1.62E-18 | -0.430789617 | 5.77E-24 |
| DCAF4L2 | 0.846034614 | 0.705617417 | -0.261830518 | 5.39E-21 | 1.70E-18 | -0.331740891 | 2.77E-14 |
| SERP2 | 0.526771656 | 0.628086523 | 0.253785593 | 6.15E-21 | 1.94E-18 | -0.578053448 | 7.94E-46 |
| TDRD1 | 0.627220404 | 0.467565929 | -0.423802687 | 8.50E-21 | 2.68E-18 | -0.784826552 | 2.39E-105 |
| BHMT2 | 0.289260751 | 0.397582363 | 0.458883175 | 9.37E-21 | 2.95E-18 | -0.611648017 | 1.57E-52 |
| ADAM28 | 0.246400566 | 0.427926853 | 0.796358646 | 1.22E-20 | 3.85E-18 | -0.441914546 | 2.86E-25 |
| KCNJ15 | 0.539082711 | 0.64583521 | 0.260659455 | 1.49E-20 | 4.71E-18 | -0.366599044 | 2.56E-17 |
| RIN1 | 0.414660922 | 0.49933913 | 0.268087874 | 1.54E-20 | 4.86E-18 | -0.406224109 | 3.00E-21 |
| DUSP6 | 0.148419587 | 0.258125517 | 0.798391266 | 1.71E-20 | 5.40E-18 | -0.38709482 | 2.76E-19 |
| TMEM129 | 0.607580573 | 0.438889814 | -0.469216953 | 1.86E-20 | 5.86E-18 | -0.490762096 | 1.32E-31 |
| GLRA4 | 0.567077746 | 0.669722289 | 0.240016441 | 2.38E-20 | 7.49E-18 | -0.374497644 | 4.64E-18 |
| TMEM79 | 0.563318056 | 0.40509796 | -0.475678896 | 2.56E-20 | 8.07E-18 | -0.454056177 | 9.46E-27 |
| ZNF492 | 0.057359216 | 0.147069493 | 1.358400807 | 3.63E-20 | 1.14E-17 | -0.334723208 | 1.58E-14 |
| KRT17 | 0.429753716 | 0.576878311 | 0.42475691 | 3.93E-20 | 1.24E-17 | -0.426950892 | 1.59E-23 |
| HPDL | 0.142490298 | 0.356177408 | 1.321732318 | 5.48E-20 | 1.73E-17 | -0.544167782 | 8.31E-40 |
| TBX4 | 0.527640716 | 0.644669755 | 0.289004405 | 5.51E-20 | 1.74E-17 | -0.378495752 | 1.92E-18 |
| CXorf65 | 0.61047736 | 0.714888485 | 0.227780422 | 6.01E-20 | 1.89E-17 | -0.345108546 | 2.11E-15 |
| RPL3 | 0.516448658 | 0.390168014 | -0.404529422 | 6.72E-20 | 2.12E-17 | -0.330229103 | 3.68E-14 |
| ALDH1A3 | 0.390659331 | 0.266302408 | -0.552845598 | 6.96E-20 | 2.19E-17 | -0.500862763 | 4.78E-33 |
| ITGB8 | 0.680353935 | 0.811428455 | 0.254178432 | 7.39E-20 | 2.33E-17 | -0.329525508 | 4.20E-14 |
| EPSTI1 | 0.290640953 | 0.488764354 | 0.749901072 | 9.37E-20 | 2.95E-17 | -0.36637029 | 2.69E-17 |
| CD74 | 0.289448145 | 0.38147965 | 0.398301191 | 9.86E-20 | 3.11E-17 | -0.401621542 | 9.15E-21 |
| LCNL1 | 0.5367385 | 0.652843569 | 0.282517967 | 1.15E-19 | 3.63E-17 | -0.340753557 | 4.95E-15 |
| ALDH3A1 | 0.305586343 | 0.415499687 | 0.44326732 | 1.48E-19 | 4.67E-17 | -0.517874541 | 1.38E-35 |
| ZNF296 | 0.488005751 | 0.609309524 | 0.32027714 | 1.57E-19 | 4.96E-17 | -0.382489357 | 7.86E-19 |
| RASSF7 | 0.58546757 | 0.43429025 | -0.430929696 | 1.88E-19 | 5.93E-17 | -0.387871484 | 2.31E-19 |
| RMI1 | 0.447530462 | 0.368365451 | -0.280848127 | 2.05E-19 | 6.46E-17 | -0.337857468 | 8.66E-15 |
| PHB2 | 0.427685674 | 0.303722572 | -0.493796752 | 2.27E-19 | 7.16E-17 | -0.477562707 | 8.62E-30 |
| GFRA3 | 0.411337002 | 0.536842603 | 0.384178311 | 2.34E-19 | 7.37E-17 | -0.360072932 | 1.01E-16 |
| TRIM68 | 0.326771428 | 0.266324758 | -0.295095295 | 3.32E-19 | 1.04E-16 | -0.320062635 | 2.38E-13 |
| PEX10 | 0.500676991 | 0.401928074 | -0.316942809 | 3.50E-19 | 1.10E-16 | -0.799072403 | 6.85E-112 |
| RTP4 | 0.10251882 | 0.273692359 | 1.416666376 | 4.91E-19 | 1.55E-16 | -0.510739286 | 1.67E-34 |
| EVX2 | 0.193208059 | 0.354341529 | 0.874985288 | 5.04E-19 | 1.59E-16 | -0.368485847 | 1.71E-17 |
| GSTM1 | 0.217137433 | 0.417699712 | 0.94385769 | 5.72E-19 | 1.80E-16 | -0.350100252 | 7.79E-16 |
| PCYT1B | 0.369422423 | 0.485039265 | 0.392830103 | 7.33E-19 | 2.31E-16 | -0.324885116 | 9.92E-14 |
| NEU1 | 0.461750757 | 0.549814578 | 0.251830834 | 1.06E-18 | 3.34E-16 | -0.627546222 | 5.40E-56 |
| SMIM10 | 0.169555334 | 0.460955131 | 1.442870156 | 1.29E-18 | 4.06E-16 | -0.520977027 | 4.58E-36 |
| MPV17L | 0.058707752 | 0.119466783 | 1.024986635 | 1.75E-18 | 5.50E-16 | -0.561841182 | 7.35E-43 |
| FAM200A | 0.052276828 | 0.163373947 | 1.643934435 | 1.78E-18 | 5.60E-16 | -0.324913822 | 9.86E-14 |
| HRASLS5 | 0.247565475 | 0.377006175 | 0.606778021 | 2.64E-18 | 8.33E-16 | -0.440807228 | 3.88E-25 |
| CFTR | 0.411105971 | 0.577945673 | 0.49142356 | 2.85E-18 | 8.97E-16 | -0.529637608 | 1.98E-37 |
| MARS | 0.661117252 | 0.407190703 | -0.69920154 | 2.88E-18 | 9.08E-16 | -0.402813221 | 6.87E-21 |
| HFE | 0.274027044 | 0.439762971 | 0.682407851 | 2.94E-18 | 9.27E-16 | -0.715909703 | 1.42E-79 |
| KRT15 | 0.435093982 | 0.561032177 | 0.366756454 | 7.23E-18 | 2.28E-15 | -0.609414077 | 4.63E-52 |
| OXA1L | 0.773996247 | 0.65576957 | -0.239137612 | 8.27E-18 | 2.61E-15 | -0.389635661 | 1.54E-19 |
| ZNF454 | 0.147959295 | 0.270635408 | 0.871150276 | 9.58E-18 | 3.02E-15 | -0.55690797 | 5.46E-42 |
| SLC14A1 | 0.608035091 | 0.759503032 | 0.320901137 | 9.85E-18 | 3.10E-15 | -0.66896223 | 4.95E-66 |
| IFITM1 | 0.356201314 | 0.463243762 | 0.379078713 | 1.10E-17 | 3.48E-15 | -0.558041691 | 3.45E-42 |
| ACP5 | 0.49004256 | 0.611975001 | 0.320565671 | 1.23E-17 | 3.88E-15 | -0.527208252 | 4.83E-37 |
| SSPN | 0.397295309 | 0.475949424 | 0.260596518 | 1.24E-17 | 3.90E-15 | -0.370168488 | 1.19E-17 |
| HOXA7 | 0.477545174 | 0.60708724 | 0.346266638 | 1.38E-17 | 4.35E-15 | -0.608091983 | 8.76E-52 |
| DHCR24 | 0.403593256 | 0.348849044 | -0.210299188 | 1.76E-17 | 5.56E-15 | -0.545036319 | 5.94E-40 |
| C1orf116 | 0.543311041 | 0.417651447 | -0.37947893 | 1.98E-17 | 6.22E-15 | -0.462437381 | 8.29E-28 |
| KCNE3 | 0.353838643 | 0.483554182 | 0.450585939 | 2.19E-17 | 6.91E-15 | -0.417977981 | 1.61E-22 |
| CYP27A1 | 0.197105263 | 0.355927575 | 0.852617408 | 2.67E-17 | 8.41E-15 | -0.678160261 | 1.74E-68 |
| NRSN2 | 0.143382441 | 0.211216393 | 0.55885345 | 2.74E-17 | 8.62E-15 | -0.749754463 | 3.56E-91 |
| GPR150 | 0.213586693 | 0.356281209 | 0.73819463 | 2.85E-17 | 8.97E-15 | -0.435830595 | 1.50E-24 |
| MEIS1 | 0.380878186 | 0.48527249 | 0.349465414 | 3.02E-17 | 9.52E-15 | -0.450427706 | 2.66E-26 |
| C19orf33 | 0.409313316 | 0.551774967 | 0.430874405 | 4.14E-17 | 1.31E-14 | -0.492480747 | 7.58E-32 |
| EPB41L3 | 0.187774807 | 0.294365945 | 0.64860726 | 4.16E-17 | 1.31E-14 | -0.362335147 | 6.30E-17 |
| SMYD4 | 0.516916119 | 0.632276474 | 0.290625349 | 8.01E-17 | 2.52E-14 | -0.357643522 | 1.67E-16 |
| ISL2 | 0.277628122 | 0.398155827 | 0.520179461 | 9.05E-17 | 2.85E-14 | -0.387250154 | 2.67E-19 |
| TRIM61 | 0.399296152 | 0.522166739 | 0.387051396 | 1.12E-16 | 3.53E-14 | -0.445295364 | 1.12E-25 |
| SLC10A5 | 0.682378547 | 0.421046571 | -0.696592477 | 1.19E-16 | 3.76E-14 | -0.320114016 | 2.36E-13 |
| RNF145 | 0.345873421 | 0.394683041 | 0.190450377 | 1.58E-16 | 4.98E-14 | -0.353804146 | 3.68E-16 |
| ACSM1 | 0.701622464 | 0.615246672 | -0.189529991 | 2.01E-16 | 6.34E-14 | -0.505238527 | 1.10E-33 |
| LGALS3 | 0.176724974 | 0.29394319 | 0.734031423 | 3.62E-16 | 1.14E-13 | -0.639932037 | 7.80E-59 |
| ORM1 | 0.850984764 | 0.890157001 | 0.06492651 | 3.88E-16 | 1.22E-13 | -0.656282137 | 8.64E-63 |
| HIST1H2BH | 0.073047428 | 0.264894343 | 1.85851166 | 1.40E-15 | 4.41E-13 | -0.371379999 | 9.15E-18 |
| CDO1 | 0.284718177 | 0.441623712 | 0.633283039 | 1.56E-15 | 4.90E-13 | -0.604909778 | 4.02E-51 |
| STK33 | 0.157789146 | 0.22921583 | 0.538708714 | 1.72E-15 | 5.40E-13 | -0.375855294 | 3.44E-18 |
| B3GNT9 | 0.25662882 | 0.363322187 | 0.501566273 | 2.23E-15 | 7.01E-13 | -0.476659502 | 1.14E-29 |
| TDRD5 | 0.327704749 | 0.424913368 | 0.374772149 | 2.54E-15 | 8.00E-13 | -0.367910131 | 1.93E-17 |
| BST2 | 0.582425042 | 0.669868938 | 0.201806468 | 3.73E-15 | 1.18E-12 | -0.604071269 | 5.98E-51 |
| ENTPD5 | 0.843080189 | 0.75806388 | -0.153350434 | 3.76E-15 | 1.19E-12 | -0.484983145 | 8.42E-31 |
| ARMCX1 | 0.399337056 | 0.502618678 | 0.331857336 | 3.82E-15 | 1.20E-12 | -0.669126605 | 4.48E-66 |
| ZDHHC1 | 0.287366064 | 0.370111633 | 0.36507078 | 6.36E-15 | 2.00E-12 | -0.499947808 | 6.49E-33 |
| DLEC1 | 0.147688168 | 0.258281552 | 0.806390346 | 9.68E-15 | 3.05E-12 | -0.386621005 | 3.08E-19 |
| MAP10 | 0.283165568 | 0.384852013 | 0.442657947 | 1.03E-14 | 3.26E-12 | -0.670790129 | 1.64E-66 |
| NKAPL | 0.457207304 | 0.562940603 | 0.300134258 | 1.19E-14 | 3.76E-12 | -0.513888146 | 5.59E-35 |
| CLDN8 | 0.556942422 | 0.387056997 | -0.524982157 | 1.24E-14 | 3.91E-12 | -0.339347771 | 6.50E-15 |
| RABGGTB | 0.480382575 | 0.357324628 | -0.426948469 | 1.30E-14 | 4.09E-12 | -0.514152455 | 5.10E-35 |
| C3orf30 | 0.778847791 | 0.709518612 | -0.134500881 | 1.36E-14 | 4.29E-12 | -0.464667107 | 4.29E-28 |
| AQP3 | 0.230499058 | 0.284442535 | 0.303376361 | 1.98E-14 | 6.22E-12 | -0.425710293 | 2.19E-23 |
| XAF1 | 0.301550058 | 0.413133226 | 0.45420958 | 2.13E-14 | 6.72E-12 | -0.388618291 | 1.95E-19 |
| CD40 | 0.34844995 | 0.46500954 | 0.416308865 | 2.26E-14 | 7.12E-12 | -0.551645281 | 4.47E-41 |
| GLUD1 | 0.307941365 | 0.218059163 | -0.497936058 | 2.33E-14 | 7.33E-12 | -0.481950234 | 2.19E-30 |
| STEAP4 | 0.325018084 | 0.244213574 | -0.412376605 | 3.11E-14 | 9.79E-12 | -0.394299183 | 5.21E-20 |
| KRT5 | 0.445476381 | 0.577871571 | 0.375399952 | 3.80E-14 | 1.20E-11 | -0.504875071 | 1.24E-33 |
| IL1RL2 | 0.486571318 | 0.593456572 | 0.286491182 | 6.84E-14 | 2.15E-11 | -0.716179874 | 1.17E-79 |
| NMI | 0.171082598 | 0.216882004 | 0.342217328 | 6.91E-14 | 2.18E-11 | -0.491949978 | 9.01E-32 |
| ME3 | 0.314349699 | 0.379546166 | 0.271904998 | 6.96E-14 | 2.19E-11 | -0.495158134 | 3.16E-32 |
| ZFP36L2 | 0.101663559 | 0.29307802 | 1.527482137 | 1.59E-13 | 5.02E-11 | -0.473824096 | 2.72E-29 |
| UAP1 | 0.946875738 | 0.850848031 | -0.154273631 | 2.15E-13 | 6.78E-11 | -0.712492477 | 1.70E-78 |
| CCDC169 | 0.11408961 | 0.26374084 | 1.208953574 | 2.34E-13 | 7.37E-11 | -0.452726068 | 1.38E-26 |
| ELL3 | 0.148369551 | 0.126171543 | -0.23380848 | 2.38E-13 | 7.50E-11 | -0.459172691 | 2.16E-27 |
| EIF1AY | 0.189226362 | 0.165745539 | -0.19114305 | 2.76E-13 | 8.69E-11 | -0.361648089 | 7.28E-17 |
| TOMM20 | 0.651029789 | 0.568869191 | -0.194626607 | 3.49E-13 | 1.10E-10 | -0.43118661 | 5.20E-24 |
| ZC3HAV1L | 0.241484875 | 0.301156572 | 0.31858091 | 8.21E-13 | 2.59E-10 | -0.360108862 | 1.00E-16 |
| CNN3 | 0.38689982 | 0.46481797 | 0.264705787 | 9.71E-13 | 3.06E-10 | -0.571670481 | 1.22E-44 |
| SLC46A3 | 0.190207297 | 0.252274115 | 0.407419593 | 1.33E-12 | 4.20E-10 | -0.373000082 | 6.44E-18 |
| MAOB | 0.452344628 | 0.571246619 | 0.336691383 | 1.50E-12 | 4.73E-10 | -0.456007352 | 5.40E-27 |
| OXGR1 | 0.303879314 | 0.428031488 | 0.494218464 | 1.57E-12 | 4.94E-10 | -0.321463396 | 1.85E-13 |
| CD38 | 0.231677969 | 0.320986609 | 0.470392249 | 2.42E-12 | 7.62E-10 | -0.656116715 | 9.50E-63 |
| FBXO27 | 0.048293561 | 0.126663061 | 1.391093113 | 3.13E-12 | 9.85E-10 | -0.644192365 | 7.67E-60 |
| ARX | 0.276857943 | 0.39633285 | 0.517566637 | 3.85E-12 | 1.21E-09 | -0.591149916 | 2.39E-48 |
| SEC14L6 | 0.244339034 | 0.331440054 | 0.439863608 | 5.67E-12 | 1.79E-09 | -0.351759317 | 5.58E-16 |
| SLC2A4RG | 0.5790893 | 0.452571559 | -0.355639917 | 3.48E-11 | 1.10E-08 | -0.436541928 | 1.24E-24 |
| SP140L | 0.410561621 | 0.505228629 | 0.299337621 | 3.60E-11 | 1.13E-08 | -0.65648662 | 7.68E-63 |
| SP5 | 0.388843543 | 0.49196113 | 0.33935455 | 6.31E-11 | 1.99E-08 | -0.530171378 | 1.63E-37 |
| LRRC9 | 0.217496674 | 0.335510389 | 0.625364105 | 6.47E-11 | 2.04E-08 | -0.407355779 | 2.28E-21 |
| CRACR2A | 0.369443486 | 0.461514528 | 0.321022372 | 2.34E-10 | 7.36E-08 | -0.685863641 | 1.30E-70 |
| ZNF334 | 0.159367342 | 0.26720218 | 0.745575754 | 3.08E-10 | 9.69E-08 | -0.607719565 | 1.05E-51 |
| AHR | 0.784466663 | 0.82044789 | 0.064699566 | 3.97E-10 | 1.25E-07 | -0.414133455 | 4.24E-22 |
| RDH5 | 0.395103983 | 0.486714801 | 0.300844258 | 6.34E-10 | 2.00E-07 | -0.437843938 | 8.70E-25 |
| ZSCAN20 | 0.319162332 | 0.415919378 | 0.382013511 | 1.18E-09 | 3.70E-07 | -0.603435804 | 8.08E-51 |
| SLC25A20 | 0.079471913 | 0.122771272 | 0.627456041 | 2.60E-09 | 8.20E-07 | -0.675910218 | 7.07E-68 |
| SYNJ2BP-COX16 | 0.412638093 | 0.309423739 | -0.415293127 | 2.60E-09 | 8.20E-07 | -0.360893148 | 8.53E-17 |
| MT1M | 0.327081126 | 0.414722433 | 0.342497574 | 5.60E-09 | 1.76E-06 | -0.329299946 | 4.38E-14 |
| N4BP2L1 | 0.183201315 | 0.236209434 | 0.366636731 | 1.46E-08 | 4.60E-06 | -0.402716335 | 7.03E-21 |
| EID3 | 0.174931427 | 0.306244749 | 0.80789561 | 2.59E-08 | 8.17E-06 | -0.541926095 | 1.97E-39 |
| RXFP4 | 0.53150331 | 0.619586503 | 0.221227041 | 3.37E-08 | 1.06E-05 | -0.40789704 | 1.99E-21 |
| HSPB1 | 0.052084211 | 0.108807775 | 1.06286364 | 9.22E-08 | 2.90E-05 | -0.34547639 | 1.96E-15 |
| APOD | 0.643309971 | 0.588650475 | -0.128102796 | 1.48E-07 | 4.65E-05 | -0.407348084 | 2.28E-21 |
| SLC35A1 | 0.895205865 | 0.841892686 | -0.08858314 | 2.26E-07 | 7.11E-05 | -0.459081219 | 2.22E-27 |
| IFNLR1 | 0.186646964 | 0.223823132 | 0.262047106 | 3.49E-07 | 0.000110077 | -0.360651147 | 8.97E-17 |
| TMEM154 | 0.366377669 | 0.414394004 | 0.177671557 | 4.37E-07 | 0.000137673 | -0.509498879 | 2.56E-34 |
| CRYZ | 0.112989483 | 0.130982065 | 0.21318078 | 1.25E-06 | 0.000394285 | -0.406181427 | 3.03E-21 |
| CDKL2 | 0.355038354 | 0.438900723 | 0.305919763 | 1.69E-06 | 0.000530917 | -0.422641949 | 4.86E-23 |
| HNF1B | 0.177070398 | 0.235240119 | 0.409811076 | 1.77E-06 | 0.0005575 | -0.564662797 | 2.30E-43 |
| BAK1 | 0.302824546 | 0.329719513 | 0.122757116 | 1.99E-06 | 0.000627509 | -0.369214367 | 1.46E-17 |
| GBP4 | 0.208052375 | 0.31129317 | 0.58132717 | 2.02E-06 | 0.000636282 | -0.323185415 | 1.35E-13 |
| ADAM32 | 0.244355159 | 0.339497821 | 0.47442275 | 2.38E-06 | 0.000749434 | -0.497095636 | 1.67E-32 |
| TRIM4 | 0.16352587 | 0.152133269 | -0.10418321 | 2.99E-06 | 0.000942026 | -0.333914513 | 1.84E-14 |
| PI15 | 0.384303633 | 0.436027134 | 0.182171305 | 3.02E-06 | 0.000952819 | -0.325651121 | 8.61E-14 |
| SLC36A4 | 0.044514749 | 0.071730689 | 0.68830707 | 4.50E-06 | 0.001417466 | -0.40253321 | 7.35E-21 |
| CYBRD1 | 0.215009998 | 0.263432526 | 0.293029736 | 5.15E-06 | 0.001620711 | -0.486870656 | 4.62E-31 |
| CLDN7 | 0.189382444 | 0.173336409 | -0.12772788 | 5.43E-06 | 0.001709545 | -0.552380826 | 3.34E-41 |
| ARMCX4 | 0.263025331 | 0.319593823 | 0.281037777 | 7.77E-06 | 0.002448442 | -0.529053887 | 2.46E-37 |
| ARMCX2 | 0.273066147 | 0.320617265 | 0.23160165 | 9.29E-06 | 0.002927217 | -0.754925897 | 4.11E-93 |
| MAGEH1 | 0.120507426 | 0.106923771 | -0.172539429 | 1.52E-05 | 0.004794549 | -0.34960988 | 8.60E-16 |
| NPY | 0.194220053 | 0.295281684 | 0.604399704 | 1.66E-05 | 0.005229384 | -0.35369056 | 3.77E-16 |
| RLN1 | 0.579076352 | 0.519993513 | -0.155259955 | 1.99E-05 | 0.006253299 | -0.556114196 | 7.51E-42 |
| ADPRH | 0.190065593 | 0.229040956 | 0.269108206 | 2.48E-05 | 0.00779884 | -0.487632822 | 3.62E-31 |
| WBP2NL | 0.5591685 | 0.502145151 | -0.155178639 | 2.88E-05 | 0.009067868 | -0.511053292 | 1.50E-34 |
| ALDH2 | 0.280063512 | 0.308088618 | 0.137591348 | 3.13E-05 | 0.009864155 | -0.558455512 | 2.92E-42 |
| PKP1 | 0.293313063 | 0.378717453 | 0.368680584 | 6.65E-05 | 0.020958477 | -0.750203435 | 2.43E-91 |
| IRX2 | 0.663591081 | 0.599252061 | -0.147131531 | 7.71E-05 | 0.024282307 | -0.612078291 | 1.27E-52 |
| BHLHA15 | 0.641645038 | 0.564565651 | -0.184634054 | 8.72E-05 | 0.027453476 | -0.587386683 | 1.30E-47 |
| ZNF502 | 0.284374546 | 0.277349667 | -0.036086342 | 9.03E-05 | 0.028429012 | -0.379223522 | 1.63E-18 |
| LYPD8 | 0.533527484 | 0.502706598 | -0.085845968 | 0.000123673 | 0.038956996 | -0.539165429 | 5.64E-39 |
