# Supplementary material for: Identification of key DNA methylation-driven genes in prostate adenocarcinoma: an integrative analysis of TCGA methylation data
Source: J Transl Med. 2019 Sep 18;17:311. doi: 10.1186/s12967-019-2065-2 (PMC6751626; doi:10.1186/s12967-019-2065-2)
Supplement: Supplementary file 2 — Additional file 2: Table S2. A gene list of 369 differentially methylated genes and 594 differentially methylated genes in GSE112047 and GSE76938. [file 12967_2019_2065_MOESM2_ESM.docx]

Table S2. A gene list of 369 differentially methylated genes and 594 differentially methylated genes in GSE112047 and GSE76938.

| GEO datasets | Differentially methylated genes |
| --- | --- |
| GSE112047 | ABHD3, ACBD4, ACSS3, ACTN2, ADAM8, ADAMTS5, AEBP1, AKR1B1, ALDH1L1, AMPH, ANGPTL2, ANKLE1, ANKRD30B, ANKRD53, AOX1, APC, APOBEC3C, APOBEC3F, APOBEC3G, AQP5, ARMCX1, ARX, BARHL2, BHLHB9, BST1, BVES, C10orf116, C11orf87, C11orf92, C14orf159, C19orf35, C1QL2, C1orf114, C20orf103, C20orf72, C2orf39, C2orf88, C3orf26, C3orf72, C5orf4, C9orf129, CACNA1A, CADM3, CAMK4, CARD11, CCAR1, CCDC67, CD200, CD34, CD44, CDH8, CDO1, CDX2, CENPK, CHODL, CHP2, CHST4, CMTM1, CMTM3, COL1A1, COL24A1, COL4A6, COL5A2, COX6B2, CPA6, CPNE4, CPNE5, CPVL, CPXM1, CR1, CRHR2, CTNNA2, CXCL14, CXCL1, CXCL5, CXCL6, CYBA, CYP27A1, DAB1, DCAF12L1, DDX25, DEPDC7, DFNA5, DHRS4L2, DKK2, DKK3, DLEU7, DLK1, DMGDH, DNAH10, DOK6, DPP10, DUOX1, DUOXA2, ECE2, EDIL3, EDNRB, EFCAB4B, EFEMP1, EFS, EGFL6, EPSTI1, EYA4, FADS2, FAIM2, FAM133A, FAM194B, FAM70A, FBLN5, FBXO27, FBXO39, FERD3L, FEZF2, FGF13, FLOT1, FLRT2, FOXL2, FREM2, GABRE, GAL3ST3, GALNTL6, GALR1, GATA4, GBP1, GBP2, GDA, GDAP1L1, GFRA3, GIPC2, GNAL, GOLSYN, GPR139, GPR56, GPRASP1, GPX3, GRIA3, GRIA4, GSTM2, GSTP1, GUCY2D, GYPC, HAAO, HAPLN3, HAPLN4, HAS3, HDAC9, HEYL, HFE, HFM1, HIF3A, HIST1H1D, HIST1H2BH, HIST1H3G, HIST1H4L, HLA-DOA, HLF, HMGCLL1, HMX2, HOPX, HOXA7, HRASLS, HSF4, ID1, IER3, IFI44L, IFIT3, IKZF1, INCA1, INSC, IRF4, JAKMIP2, JPH2, KCNB2, KCNH4, KCNH7, KCNJ3, KCNK2, KCNV1, KIF5A, KLF8, KLHL3, KLK10, KLK13, KRBA1, KY, LAYN, LHFPL4, LOC100130691, LOC145663, LOC283392, LOC541471, LOC644538, LRRC25, LRRC3B, LTBP3, LTC4S, MAOB, MAP7D2, 10-Mar, MCART6, MDFI, MEIS1, MEIS2, MIR1258, MIR181C, MIR9-1, MMP25, MPI, MSC, MYO3A, NBR1, NCAN, NCDN, NEFL, NEU1, NEUROG3, NFE2L3, NID2, NIPAL4, NKAIN3, NKX2-4, NKX2-5, NOX4, NPHS2, NRG1, NRIP2, NRSN2, NTNG1, NUDT16, NYNRIN, OLIG2, PAH, PAX7, PCDH11Y, PCDH8, PCDHA7, PCDHAC1, PCDHAC2, PCDHGA4, PCSK1, PDGFD, PDK4, PDZD7, PHOX2A, PHYHD1, PIP5KL1, PKP1, PLAUR, PLTP, POM121L2, POU4F1, POU4F2, PPP2R2B, PRKCB, PRKRA, PROM1, PRRT3, PRSS12, PTCH2, PTN, PUS3, RAB37, RAD51AP2, RARA, RARB, RARRES1, RASL12, RBMS3, RGS19, RHCG, RNF152, RNF175, RNU5E, RPRML, RTP4, RUNX3, SACS, SALL1, SALL2, SCGB3A1, SCRN1, SCT, SCTR, SECTM1, SERP2, SERPINB9, SERPINE1, SH3KBP1, SHROOM4, SIRPA, SLC12A5, SLC16A12, SLC19A3, SLC26A4, SLC34A2, SLC40A1, SLC43A3, SLC44A4, SLC5A7, SLC7A14, SLC7A3, SORCS3, SPARC, SPATA6, SPATS1, SPDYA, SPHKAP, SPRED3, SRPK2, SST, ST6GALNAC3, ST8SIA1, STEAP4, STK32B, STK33, SV2A, SYCP2L, SYN1, SYT10, SYT14, TAC1, TACC2, TCTEX1D1, TFPI2, TFPI, THBS1, TMEM106A, TMEM155, TMEM176B, TMEM22, TMEM35, TMEM90B, TMLHE, TNFRSF10C, TNFSF11, TPM4, TRH, TRIM7, TRIP6, TRO, TRPA1, TTLL9, UCHL1, USP2, VGLL2, VIM, VSX2, VWA3B, VWC2, VWDE, WDR69, WFDC2, WNT3, WT1, XKR7, ZFP36L2, ZNF154, ZNF211, ZNF215, ZNF334, ZNF454, ZNF492, ZNF577, ZNF655, ZNF660, ZNF804A, ZNF90, ZSCAN12, ZSWIM2 |
| GSE76938 | ABHD3, ACBD4, ACSF2, ACSS3, ADAM32, ADAM8, ADAMTS5, ADCY9, AEBP1, AIFM2, AKR1B1, ALDH1L1, AMDHD1, ANGPTL2, ANKLE1, ANKRD53, ANO5, AOX1, APC, APOBEC3C, APOBEC3F, APOBEC3G, AQP5, ARMCX1, ARX, ASTN1, ATP2B4, BARHL2, BCAT2, BEX1, BHLHB9, BHLHE22, BHLHE41, BTG1, BTG4, BVES, C10orf116, C11orf87, C11orf92, C11orf93, C13orf38, C14orf159, C17orf46, C18orf1, C19orf35, C1QL2, C1orf114, C1orf51, C1orf94, C20orf166, C2orf39, C2orf88, C3orf18, C3orf26, C3orf62, C3orf72, C5orf4, C6orf103, C6orf114, C6orf141, C7orf31, C9orf110, C9orf129, C9orf68, CA3, CACNA1A, CADM3, CAMK4, CARD11, CAST, CBLN1, CCBE1, CCDC65, CCDC67, CCDC88A, CD200, CD34, CD44, CDC42EP5, CDH13, CDH18, CDH8, CDKL2, CDKN2A, CDO1, CDX2, CFTR, CHAT, CHP2, CHST4, CIDEA, CITED4, CLDN5, CMBL, CMTM3, CNN3, COL14A1, COL1A2, COL3A1, COL4A6, COL5A2, CORIN, COX6B2, CPA6, CPNE4, CPNE5, CPT1C, CPVL, CPXM1, CR1, CRH, CRHR2, CSGALNACT1, CTNNA2, CTSA, CTSS, CUGBP2, CXCL14, CXCL2, CXCL5, CYBA, CYBRD1, CYP1B1, CYP27A1, CYYR1, DAB1, DAP3, DCAF12L1, DCC, DCHS2, DDX25, DEPDC7, DERL3, DFNA5, DGKZ, DHRS4L2, DKK1, DKK2, DKK3, DLK1, DMGDH, DMRTA2, DNAH10, DOK5, DPP10, DPYS, DUOX1, DUOXA2, ECE2, ECHDC3, EDIL3, EDNRB, EFCAB4B, EFEMP1, EFS, EGFL6, EIF1B, EMP3, EPB41L3, EPHA10, EPHA3, EPSTI1, EREG, ESRRG, ESX1, EYA1, EYA4, FADS2, FAIM2, FAM133A, FAM149A, FAM162B, FAM194B, FAM70A, FAM7A3, FBLN5, FBXL21, FBXO39, FERD3L, FEZF2, FGF12, FGF13, FLJ30058, FLJ45983, FLRT2, FN1, FOXN4, FRZB, FUT9, GABRA2, GABRA3, GABRA4, GABRB1, GABRE, GABRG2, GADD45A, GAL3ST3, GALNT9, GALNTL6, GALR1, GATA4, GBP1, GBP2, GDAP1L1, GDF10, GDPD5, GFM1, GFRA1, GFRA3, GIPC2, GLIPR1, GNAL, GNG4, GPR139, GPR149, GPR56, GPRASP1, GPX3, GRIA1, GRIA3, GRIA4, GRIN2A, GRM3, GRM6, GSTM2, GSX1, GUCY2D, GYPC, HAAO, HAPLN3, HAPLN4, HAS2, HAS3, HBQ1, HCRTR2, HEYL, HFE, HFM1, HIF3A, HIST1H1A, HIST1H1B, HIST1H1D, HIST1H2BH, HIST1H2BI, HIST1H3G, HIST1H3J, HIST1H4D, HIST1H4L, HLA-DOA, HLF, HMGCLL1, HMX2, HOPX, HOXA7, HPDL, HRASLS5, HRASLS, HSF4, HSPA9, HSPB1, HTR1E, IER3, IFI27L2, IFI44L, IFIT3, IGF2BP1, IKZF1, IL1B, IL1RAPL2, INCA1, INSC, IRAK3, IRF4, JAKMIP2, JPH2, KCNB2, KCNC4, KCND1, KCNE3, KCNH4, KCNH7, KCNJ3, KCNJ8, KCNK2, KCNQ5, KCNV1, KIAA1217, KIF19, KIF5A, KIRREL, KLF8, KLHL3, KLK10, KLK13, KLK5, KRBA1, KRT222, KY, LAMA2, LAYN, LDB1, LGALS1, LIG4, LOC100286793, LOC145663, LOC283856, LOC344595, LOC401093, LOC401097, LOC541471, LOC644538, LRP1B, LRRC17, LRRC25, LRRC3B, LTBP3, LY75, MAGI2, MAOB, MAP7D2, MAPT, 10-Mar, MARVELD1, MCC, MDFI, ME1, MEIS1, MEIS2, MIR1258, MIR129-2, MIR181C, MIR301B, MIR375, MIR548N, MIR9-1, MLLT11, MME, MMP23B, MPP2, MT1E, MYO3A, NAALAD2, NAV2, NBR1, NCAN, NCRNA00152, NCRNA00188, NEAT1, NEFL, NELL1, NEU1, NEUROD1, NEUROG3, NID2, NIPAL4, NKAIN3, NKX2-1, NKX2-4, NKX2-5, NLGN1, NMNAT3, NOX4, NPAS4, NPHS2, NPY1R, NPY, NR0B1, NRG1, NRIP2, NRK, NRSN2, NRXN3, NTNG1, NUAK1, NYNRIN, OLIG2, OPRM1, PAH, PAK3, PALM2-AKAP2, PAMR1, PANK1, PAX3, PAX7, PCBP1, PCDH11Y, PCDH8, PCDHA7, PCDHAC1, PCDHAC2, PCDHGA4, PCDHGA5, PCSK1, PCYT1B, PDE1C, PDGFD, PDK4, PDLIM4, PDZD7, PGR, PHOX2A, PHYHD1, PIP5KL1, PKP1, PLCD4, PLCE1, PLEC1, PLTP, POSTN, PPARGC1A, PPM1M, PPP1R3C, PPP2R2B, PPT2, PRKCB, PRKRA, PROM1, PRSS12, PTCH2, PTGDR, PTGS2, PTN, PTPRO, PTX3, PUS3, QRFPR, RAD51AP2, RARA, RARB, RARRES1, RASAL2, RASL12, RBM9, RBMS3, REM1, RGN, RGS19, RNF152, RNF175, ROBO1, RTP4, RUNDC3A, RUNX3, RUSC2, SALL1, SALL2, SAMD11, SCGB3A1, SCRN1, SCT, SCTR, SDPR, SECTM1, 4-Sep, 9-Sep, SERP2, SERPINB9, SERPINE1, SERPING1, SETBP1, SFRP2, SFRP5, SH3BGRL, SH3KBP1, SHISA9, SHROOM4, SIRPA, SLC16A12, SLC16A13, SLC18A3, SLC19A3, SLC25A22, SLC34A2, SLC38A4, SLC40A1, SLC43A3, SLC44A4, SLC5A7, SLC7A14, SLC7A3, SLCO4C1, SLITRK2, SNHG3-RCC1, SNHG9, SNORD123, SORBS2, SORCS3, SOSTDC1, SPAG17, SPARC, SPATA6, SPATS1, SPDYA, SPHKAP, SPRED3, SRPK2, SRY, SST, ST6GALNAC3, ST8SIA1, ST8SIA3, ST8SIA4, STK32B, STK33, STMN2, STXBP6, SV2A, SYCP2L, SYN1, SYT10, SYT14, SYT6, SYT9, TAC1, TCEAL5, TCTEX1D1, TEKT2, TEKT3, TF, TFPI2, TFPI, TGFB1, THBS1, THBS4, THY1, TLX1NB, TMED7-TICAM2, TMEM106A, TMEM130, TMEM155, TMEM176B, TMEM178, TMEM199, TMEM215, TMEM220, TMEM35, TMLHE, TNFAIP8, TNFRSF10C, TRAPPC9, TRH, TRIB2, TRIM7, TRIP6, TRO, TRPA1, TRPC5, TRPM3, TTBK1, TTLL9, TUBA4B, TULP1, TXNRD2, UCHL1, UFSP1, USP2, VCAM1, VCAN, VGLL2, VIM, VSNL1, VSTM2A, VSX2, VWA3B, VWA5A, VWC2, WDR69, WDR86, WFDC2, WNT10A, WNT2, WNT3, XKR7, XKRX, ZDHHC15, ZFP36L2, ZNF154, ZNF215, ZNF219, ZNF233, ZNF334, ZNF385B, ZNF454, ZNF492, ZNF577, ZNF578, ZNF642, ZNF655, ZNF660, ZNF662, ZNF804A, ZNF90, ZSCAN12, ZSWIM2 |
